# Tracking spatio-temporal dynamics of early immune responses to an intranasal OMV-based pneumococcal vaccine candidate in mice

**DOI:** 10.1038/s41541-026-01430-y

**Published:** 2026-03-30

**Authors:** Sajida Kanwal, Shaina Vivienne To, Rienke Uijen, Rona Roverts, Bram van Cranenbroek, Fred J. van Opzeeland, Ben Joosten, Bart van den Berg van Saparoea, Christa E. van der Gaast-de Jongh, Esther van Rijssen, Joen Luirink, Dimitri A. Diavatopoulos, Lucille F. van Beek, Marien I. de Jonge

**Affiliations:** 1 Department of Laboratory Medicine, Laboratory of Medical Immunology, Radboud University Medical Center, Radboudumc Community for Infectious Diseases, Nijmegen, The Netherlands; 2 Electron Microscopy Center, Radboud University Medical Center, Nijmegen, The Netherlands; 3 Abera Bioscience AB, Uppsala, Sweden

**Keywords:** Immunology, Microbiology

## Abstract

Outer membrane vesicle (OMV)-based vaccines elicit strong immune responses and have emerged as a versatile platform for targeting multiple pathogens, yet the mechanisms underlying their efficacy remain incompletely understood. Here, we investigated the early immune response following intranasal administration of an OMV-based pneumococcal vaccine candidate in mice. Using in vivo bioluminescence imaging, spectral flow cytometry, and high-resolution microscopy, we tracked OMV biodistribution and immune activation throughout the respiratory tract from 1 to 72 h post-immunization. OMVs persisted in the nasal cavity for up to 48 h and rapidly recruited Ly6G^hi^ neutrophils and myeloid-derived suppressor cells, followed by activation of local T cells. MHCII expression was significantly upregulated on Ly6G^hi^ neutrophils in nasal tissue and coincided with a marked expansion at 24 h. In the lungs, alveolar macrophages and plasmacytoid dendritic cells emerged as early responders. OMV exposure also induced costimulatory molecule expression across multiple myeloid cell subsets. Together, these findings reveal distinct spatio-temporal patterns of innate and adaptive immune activation at mucosal sites, providing mechanistic insight into OMV-induced mucosal immunity and underscoring their potential as a versatile vaccine platform.

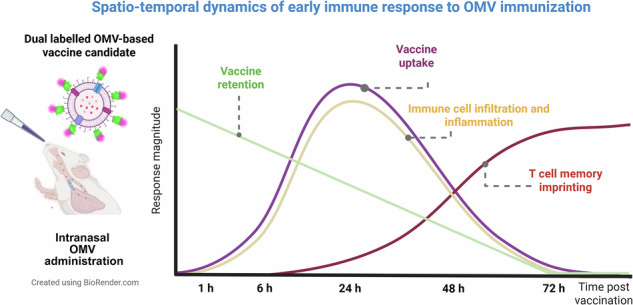

## Introduction

Outer membrane vesicles (OMVs) are nanoscale particles naturally released by Gram-negative bacteria and are increasingly recognized as potent and versatile vaccine platforms^1^. These vesicles naturally package pathogen-associated molecular patterns^2^, enabling efficient uptake by antigen-presenting cells and robust activation of innate immunity through receptors such as TLR2, TLR4, and NOD-like receptors^3,4^. Beyond their intrinsic adjuvanticity, OMVs can be engineered to display heterologous antigens, providing customizable carriers for subunit vaccine candidates^5^.

The licensure of an OMV-based vaccine for *Neisseria meningitidis* has demonstrated the translational feasibility of this approach and inspired similar strategies for other pathogens using various administration routes^6^. However, the early immune dynamics elicited by intranasal immunization with OMV-based vaccine candidates remain poorly defined, particularly the role of innate subsets such as neutrophils, monocytes and dendritic cells, which are likely to shape ensuing adaptive immune responses^7,8^.

The respiratory mucosa, a major entry site for many pathogens, is equipped with specialized innate and adaptive networks that balance rapid microbial clearance with preservation of tissue integrity^9,10^. As the body’s first line of immune protection, the mucosal immune system can mount robust local and systemic responses through coordinated cellular and molecular pathways^9,10^ while maintaining tolerance to harmless stimuli^10^. Intranasal vaccination directly engages this compartment, offering needle-free administration, enhanced patient compliance, and the potential to elicit both mucosal and systemic immunity^11^. Indeed, intranasal subunit vaccines have been shown to induce strong mucosal IgA production and systemic antibody responses, highlighting the respiratory mucosa as a strategic target for vaccine delivery^12^. Yet, how OMVs engage these mucosal immune networks during the earliest stages after vaccination remains largely unknown.

To address this, we investigated the spatio-temporal immune cascade following intranasal immunization with OMVs. OMVs derived from *Salmonella enterica* serovar Typhimurium were engineered to display conserved *Streptococcus pneumoniae* antigens (AliA and PnrA) fused to Nano luciferase (nLuc)^13,14^ for longitudinal in vivo imaging. The individual pneumococcal antigens i.e., AliA and PnrA^15,16^ as well as OMVs harboring these antigens have previously been shown to confer protection against pneumococcal colonization in vivo^17,18^. Building on this established protective efficacy, biodistribution and immune recruitment were mapped across the nasal tissue, lungs, draining lymph nodes, and spleen during the first 72 h after immunization. In addition, targeted correlative light and electron microscopy (CLEM) combined with volumetric focused ion beam–scanning electron microscopy (FIB-SEM) was performed on nasal tissue at 24 h post-immunization to visualize OMV-immune cell interactions at subcellular resolution.

**Fig. 1:**
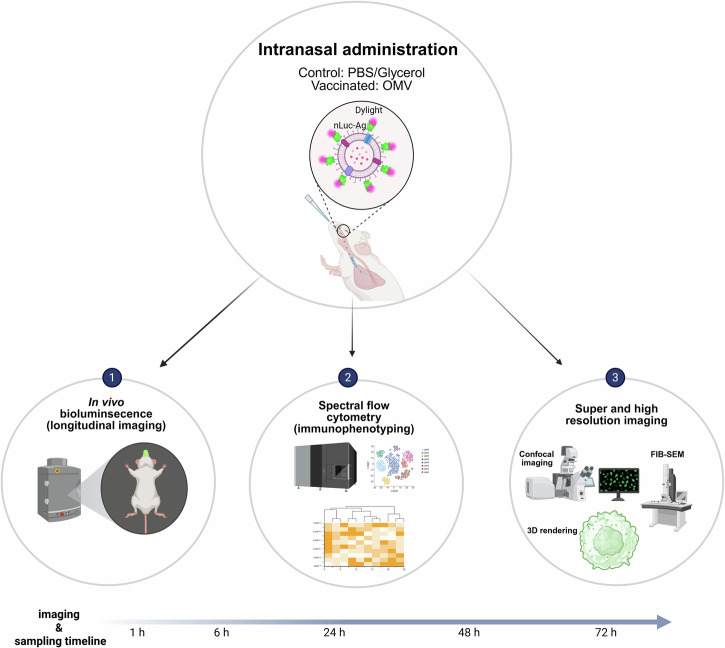
Multimodal experimental workflow for assessing immune responses to intranasal OMV-based immunization. Mice were intranasally administered with OMV-based vaccine particles engineered to display pneumococcal antigens fused to Nano luciferase (nLuc) and labelled with a fluorescent dye. Three complementary approaches were employed to evaluate vaccine retention, biodistribution, and immune responses at multiple timepoints (1, 6, 24, 48, and 72 h post-immunization): (1) In vivo bioluminescence imaging enabled longitudinal tracking of vaccine particles; (2) spectral flow cytometry for high-dimensional immune profiling and phenotypic clustering; and (3) super-resolution and ultrastructural imaging, including confocal microscopy and FIB-SEM, to visualize cellular uptake and subcellular localization of vaccine particles. The image was created via BioRender.

By combining longitudinal imaging with high-dimensional immune profiling and ultrastructural analysis, we characterized the kinetics of OMV biodistribution, identified the immune subsets directly engaging vaccine particles, and visualized their uptake at subcellular resolution (Fig. 1). These data establish a framework for dissecting early mucosal vaccine responses and highlight both classical and unconventional pathways of antigen handling that can inform the rational design of next-generation intranasal vaccines.

## Results

### Intranasal OMV-based vaccine particles are retained in the upper airways and disseminate to the lower respiratory tract within 48 h

**Fig. 2:**
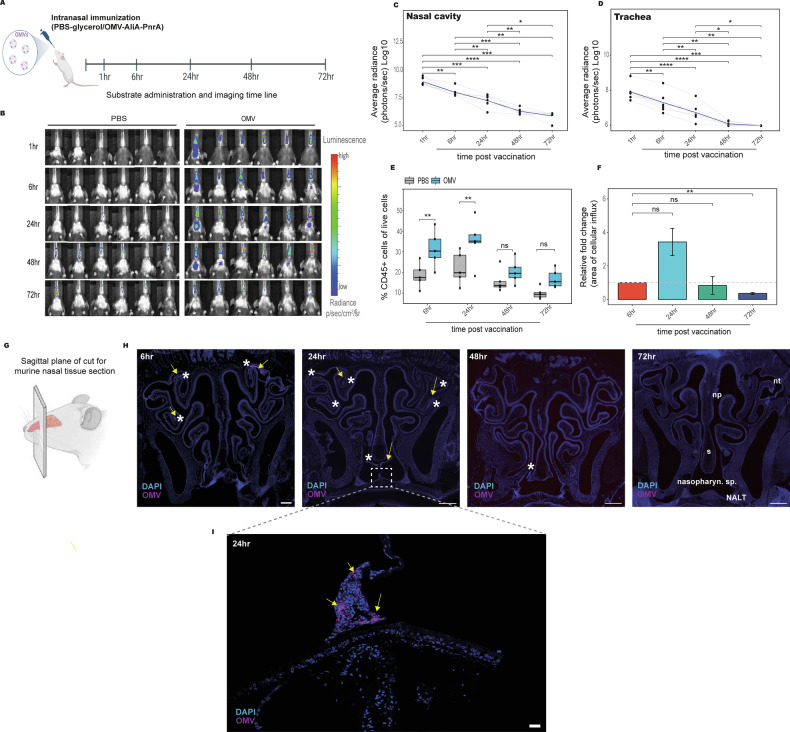
OMV-based vaccine particles persist in the nasal cavity and drive early immune cell influx in the upper airways. **A** Graphical representation of immunization and imaging timeline. **B** Representative in vivo bioluminescence images showing the retention and biodistribution of furimazine-activated nLuc-OMV particles in mice. Mice were intranasally administered with OMV-based pneumococcal vaccine candidate (5 µL); at defined time points post immunization, furimazine substrate (10 µL) was delivered intranasally and bioluminescence captured using IVIS imaging (*n* = 6). Exposure time was optimized by the IVIS system based on the preferential input for optimal luminescence. **C**, **D** Line graphs depicting the retention kinetics of nLuc-OMV particles in the nasal cavity and trachea over time. Regions of interest (ROI) were defined, and radiance (photons/second) was quantified using Living Image software, Revvity, version 3.2. Mean background signal from PBS-treated controls was subtracted (OMV - PBS). The thick blue line indicates the mean signal; grey lines represent values from individual animals (*n* = 6 mice). Statistical analysis was performed using repeated measures ANOVA. **E** Boxplots showing the proportion of CD45⁺ immune cells in live cells in nasal tissue across treatment groups and time points. Pairwise statistical comparisons between PBS and OMV groups were performed using either an unpaired *t*-test or Wilcoxon rank-sum test following Shapiro-Wilk normality testing (*n* = 5 mice per group). **F** Bar plot comparing the quantified area of immune cell influx in the murine nasal cavity at each time point post immunization, relative to the 6 h baseline. Pairwise *t*-tests were used to compare mean infiltrate area at each time point to the 6 h reference. **G** Sagittal plane showing the direction of the nasal tissue sections obtained for microscopy. **H** Representative cross-sections of murine nasal cavity showing spatial colocalization of fluorescently labeled vaccine particles (DyLight650, magenta) and immune infiltrates (DAPI, blue) at defined time points post intranasal immunization. Asterisks mark the location of immune infiltrates; yellow arrows indicate vaccine particles. Scale bar = 2 mm. np nasal passage, nt nasal turbinates, NALT nasal-associated lymphoid tissue, nasopharyng. sp. nasopharyngeal space, S septum. **I** Magnified view of translocation of vaccine particles and immune infiltrates towards the nasopharyngeal space adjacent to nasal associated lymphoid tissue at 24 h post-immunization. Images acquired by LSM900, 63x tilescan. Scale bar=10 µm. Significance stars indicate *p*-values as follows: * *p* ≤ 0.05; ** *p* ≤ 0.01; *** *p* ≤ 0.001; ns not significant.

To track the in vivo biodistribution of an intranasally delivered OMV-based vaccine against *S. pneumoniae*, we engineered *S. enterica* serovar Typhimurium-derived OMVs displaying the pneumococcal antigens AliA and PnrA fused to nLuc. Bioluminescence was monitored using an in vivo imaging system (IVIS) (Fig. 2A). Luminescent signals in the nasal cavity were observed as early as 1 h post-immunization and extended towards the thoracic region (Fig. 2B), consistent with dissemination to the lower respiratory tract. Signal intensity was detectable at both sites up to 48 h and no longer visible by 72 h (Fig. 2C, D).

To assess immune cell recruitment at the vaccine administration site, we analyzed nasal tissues by flow cytometry at defined time points post-immunization. Immunized mice exhibited a marked increase in CD45⁺ cells compared with PBS-treated controls, rising to 31% at 6 h (*p* = 0.03), peaking at 35% at 24 h (*p* = 0.06), and declining to 17% by 72 h (*p* = 0.019), consistent with the kinetics of vaccine clearance (Fig. 2E, Supplementary fig. 1).

These findings were supported by fluorescence microscopy of 5 µm microtomed sagittal sections from murine nasal tissue (Fig. 2G). DAPI-stained immune infiltrates and fluorescently labeled OMV particles were visible within the nasal turbinates and passages at 6 h post-immunization (Fig. 2H). Quantification of the infiltrated area (Supplementary fig. 2) showed a 3.4-fold expansion at 24 h relative to 6 h (*p* = 0.09) and a subsequent decline at 72 h (0.3-fold relative to 6 h, *p* = 0.008) (Fig. 2F). At 24 h post-immunization, both immune infiltrates and OMV particles extended into the nasopharyngeal region adjacent to the nasal-associated lymphoid tissue (NALT) (Fig. 2I).

### OMV-based mucosal immunization induces a spatio-temporal reshaping of the innate immune compartment

**Fig. 3:**
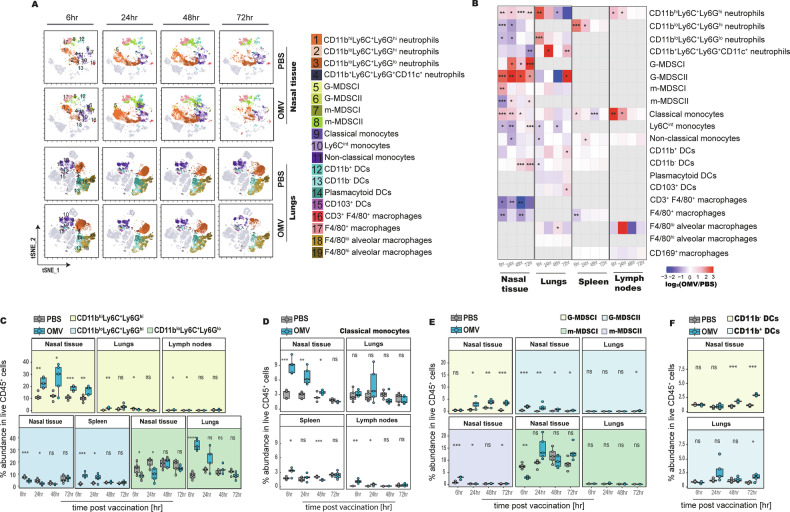
Spatio-temporal dynamics and tissue distribution of myeloid cell populations following intranasal OMV-based pneumococcal immunization. **A** Representative t-SNE plots showing the distribution of myeloid cell populations identified in murine nasal tissue and lungs across different time points post-immunization with OMVs or PBS-glycerol. Phenotypes were identified by unsupervised FlowSOM analysis (Cytobank) on live CD45⁺ cells. **B** Heatmap showing the log₂ fold change in abundance of myeloid cell populations across nasal tissue, lungs, spleen, and lymph nodes post-immunization (*n* = 5). Fold changes were calculated by dividing OMV-vaccinated values by PBS controls, log₂-transformed, and capped at −3 and +3 to enhance visualization. Grey boxes indicate the absence of respective population in respective tissues. **C**–**F** Boxplots depicting the kinetics of abundance of distinct myeloid populations in nasal tissue, lungs, spleen, and draining lymph nodes over time (*n* = 5 mice per time point). The central line indicates the median; box limits represent the interquartile range (IQR); whiskers extend to data points within 1.5× IQR, and points beyond this range are shown as outliers. Pairwise comparisons between PBS and OMV groups were performed using a t-test or Wilcoxon rank-sum test, following Shapiro-Wilk normality testing (*n* = 5 mice per group). Significance stars indicate *p*-values as follows: * *p* ≤ 0.05; ** *p* ≤ 0.01; *** *p* ≤ 0.001; ns not significant. G-MDSC granulocytic myeloid derived suppressor cell, m-MDSC monocytic myeloid derived suppressor cell, DC dendritic cell.

To characterize early innate immune responses to intranasal OMV-based pneumococcal vaccine candidate, we performed high-dimensional spectral flow cytometry (31-marker panel) on nasal tissue, lungs, nasal draining lymph nodes, and spleen (Supplementary Table 1, Supplementary figs. 3–6). Unsupervised clustering using Cytobank^19^ and FlowSOM^19^ identified 20 myeloid populations spanning five major lineages: neutrophils, monocytes, dendritic cells (DCs), macrophages, and myeloid derived suppressor cells (MDSC)-like cells (Fig. 3A, Supplementary fig. 7). To visualize the distribution and magnitude of spatio-temporal shifts in these phenotypes, we generated a heatmap of log₂ (OMV/PBS) fold changes across all tissues (Fig. 3B).

In the nasal tissue, OMVs triggered a rapid influx of CD11b^hi^Ly6C^+^Ly6G^hi^ neutrophils (1.9 fold-change [FC] compared to PBS-treated mice, *p* = 0.007) and Ly6C^hi^ inflammatory/classical monocytes (2.8 FC vs PBS, *p* = 0.0003) as early as 6 h post-immunization. These populations peaked at 24 h and declined by 72 h (Fig. 3B, D). Similar kinetics were observed in the lungs, where neutrophil subsets increased significantly at 6 h (Ly6G^hi^: 6.2 FC vs PBS, *p* = 0.002; Ly6G^lo^: 3.7 FC vs PBS, *p* = <0.0005), followed by an increase in classical monocytes at 24 h (1.9 FC vs PBS, p = 0.2). Comparable trends were seen in the draining lymph nodes and spleen (Fig. 3C, E). Neutrophil phenotype varied by tissue: in the lungs, CD11b^lo^Ly6C^+^Ly6G^lo^ neutrophils were predominant at 6 h post-immunization (34% of total neutrophils), whereas CD11b^hi^Ly6C^+^Ly6G^hi^ neutrophils represented only 2%. In the spleen, CD11b^lo^Ly6C^+^Ly6G^hi^ neutrophils showed a transient surge at 6 h (4.6 FC vs PBS, *p* = 0.0005) and 24 h (2.6 FC vs PBS, *p* = 0.04).

We also detected a heterogenous group of myeloid populations co-expressing CD11b, Ly6C, Ly6G, F4/80, and CD11c, consistent with MDSC-like phenotypes (Fig. 3A, B). Based on Ly6G expression, these were classified as granulocytic (G-MDSC) or monocytic (m-MDSC), and further subdivided into four subsets: G-MDSCI (CD11b^+^Ly6C^+^Ly6G^+^F4/80^+^CD11c^+^), G-MDSCII (CD11b^+^Ly6C^+^Ly6G^+^F4/80^+^CD11c^−^), m-MDSCI (CD11b^+^Ly6C^+^Ly6G^-^F4/80^+^CD11c^-^), and m-MDSCII (CD11b^+^Ly6C^+^Ly6G^-^F4/80^+^CD11c^+^) (Figs. 3A, B, Supplementary fig. 3). In the nasal tissue, all four subtypes were detected with distinct temporal kinetics. G-MDSCII expanded sharply at 6 h (5.5 FC vs PBS, *p* < 0.0005) and peaked at 24 h (43 FC vs PBS, *p* = 0.007), whereas G-MDSCI accumulated gradually, peaking at 72 h. m-MDSCI showed a transient spike at 6 h (3 FC vs PBS, *p* = 0.007) but not later, while m-MDSCII increased steadily from 24 h to 72 h (Fig. 3B, E). Although m-MDSCI showed a transient increase at 6 h post-immunization in comparison to samples from PBS treated animals, its relative proportion among total CD45⁺ cells did not increase to the same extent, consistent with the broader expansion of other infiltrating leukocyte populations. In the lungs, MDSC infiltration was less pronounced, although G-MDSCII and m-MDSCI populations showed detectable influx at 72 h and 24 h, respectively (Fig. 3B, E).

Dendritic cells, critical for initiating adaptive immune responses, also showed tissue- and time-specific dynamics. CD11b^-^ DCs gradually increased in the nasal tissue from 48 h (1.85 FC vs PBS, *p* < 0.0005) to 72 h (2.6 FC vs PBS, *p* = < 10⁻⁷) onward, while CD11b^+^ DCs expanded in the lungs between 24 h (2.3 FC vs PBS, *p* = 0.12) and 72 h (2 FC vs PBS, *p* < 0.027) (Fig. 3B, F).

Taken together, this analysis revealed strong enrichment of neutrophil subsets and classical monocytes in both respiratory and lymphoid tissues, with MDSC subtypes largely restricted to the nasal tissue and lungs, and selective accumulation of DCs in the respiratory tract. These data show that OMV-based mucosal immunization elicits a rapid and tissue-specific reshaping of the innate immune landscape.

### OMV-based mucosal immunization induces distinct activation of myeloid cells in both respiratory mucosa and lymphoid tissues

**Fig. 4:**
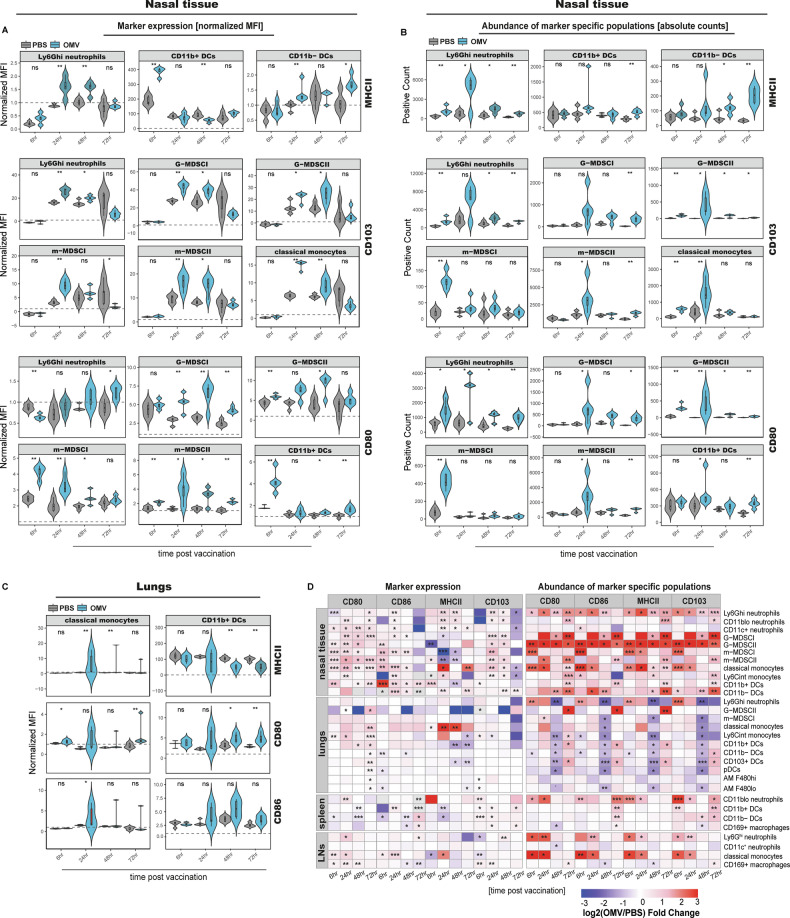
Activation states of myeloid cell subsets across respiratory and lymphoid tissues following intranasal administration of an OMV-based pneumococcal vaccine candidate. Violin plots illustrating the distribution and temporal dynamics of marker expression in nasal tissue (**A**), the distribution and temporal dynamics of abundance of marker specific populations in nasal tissue (**B**), and the distribution and temporal dynamics of marker expression in lungs (**C**). Each violin shows individual data points with an embedded box plot indicating median and interquartile range (*n* = 5 mice per time point); MFI values were normalized to tissue-specific FMO thresholds (threshold = 1, dashed line). A value > 1 indicates biologically positive expression. **D** Heatmap showing the log₂ fold change in (1) expression intensity of activation markers and (2) abundance of marker-positive myeloid subsets across nasal tissue, lungs, spleen, and lymph nodes at defined time points post-immunization. Values are log₂-transformed, capped at −3 and +3 for visualization, and represent means from *n* = 5 mice per time point and condition; grey boxes indicate marker expression below the fluorescence cutoff threshold. Pairwise comparisons between PBS and OMV groups were performed using a t-test or Wilcoxon rank-sum test, following Shapiro-Wilk normality testing (*n* = 5 mice per group). Significance stars indicate p-values as follows: * *p* ≤ 0.05; ** *p* ≤ 0.01; *** *p* ≤ 0.001; ns not significant.

To comprehensively assess the activation states of innate immune subsets across tissues, we analyzed both the mean fluorescence intensity (normalized mean fluorescence intensity and corresponding log₂ fold change (OMV/PBS)) and abundance of marker-expressing cells (absolute marker specific positive counts and corresponding log₂ fold change (OMV/PBS)) of four key activation markers i.e., CD80, CD86, MHCII, and CD103 in 20 myeloid populations across nasal tissue, lungs, spleen, and lymph nodes following intranasal immunization with the OMV-based vaccine candidate (Fig. 4A–D).

A particularly striking observation was the upregulation of MHCII on Ly6G^hi^ neutrophils in the nasal tissue in comparison to the samples from PBS treated animals (log₂ FC = 0.55, *p* = 0.02). The upregulation of MHCII by these populations in nasal tissue was accompanied by a pronounced corresponding expansion in the abundance of these populations at 24 h (nasal tissue Ly6G^hi^: log₂ FC = 2.59), reinforcing the overall shift towards an activated phenotype (Fig. 4A, B, D).

In the nasal tissue, CD103 expression was broadly upregulated across multiple myeloid subsets, including neutrophils (Ly6G^hi^: log₂ FC peak = 0.6, *p* = 0.007), MDSCs (G-MDSCI: log₂ FC = 0.65, *p* < 0.005), and monocytes (classical monocytes: log₂ FC = 1.25, *p* = 0.007), peaking at 24 h and gradually declining through 72 h (Fig. 4A, D).

Among dendritic cells, CD11b⁺ DCs in the nasal tissue exhibited early and robust activation with significant upregulation of CD80 (log₂ FC = 1.22, *p* = 0.007), CD86 (log₂ FC = 2.92, *p* < 0.005), and MHCII (log₂ FC = 1.04, *p* < 0.005) at 6 h post-immunization, notably without accompanying change in population abundance (Fig. 4A, B, D). In contrast, CD11b⁻ DCs displayed a delayed activation profile, with increase in expression of MHCII from 24 h to 72 h (log₂ FC up to ≈ 0.67, *p* = 0.006) (Fig. 4A, D), alongside a steady rise in population abundance (Fig. 4B, D).

In the lungs, the dominant activation profile was observed within the monocyte compartment. Classical monocytes exhibited a trend in marked upregulation of CD80 (log₂ FC = 1.29, *p* = 0.15), CD86 (log₂ FC = 1.2, *p* = 0.15), and MHCII (log₂ FC = 2.9, *p* = 0.007), peaking variably at 24, 48, or 72 h depending on marker and subset. Interestingly, CD11b⁺ DCs in the lungs showed a progressive decrease in MHCII expression over time (log₂ FC = –0.26 (*p* = 0.16) to –1.14 (*p* = 0.001)), contrasting with their nasal counterparts (Fig. 4C, D).

Collectively, these findings reveal a dynamic and compartmentalized pattern of myeloid cell activation and population shifts across mucosal and lymphoid tissues following intranasal OMV immunization.

### Intranasal OMV administration drives spatio-temporal programming of memory T cells

**Fig. 5:**
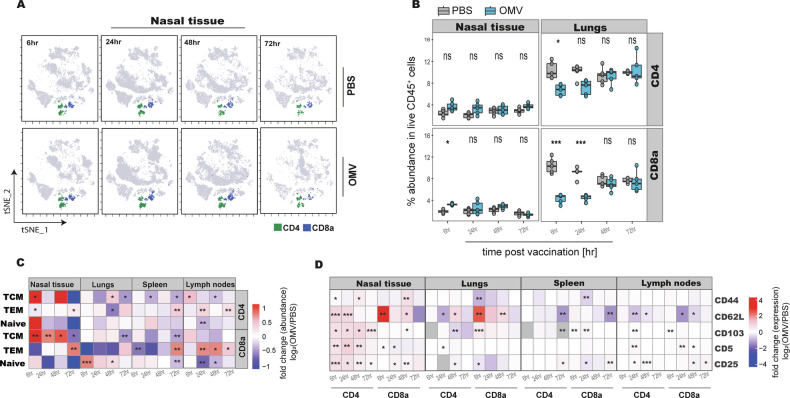
Spatio-temporal dynamics and activation states of CD4⁺ and CD8a⁺ T cells in respiratory and lymphoid tissues following intranasal OMV-based immunization. **A** Representative t-SNE plot showing the abundance of CD4⁺ and CD8a⁺ T cells in nasal tissue across different time points post immunization. Clusters were identified by unsupervised FlowSOM analysis on live CD45⁺ cells. **B** Boxplots depicting the kinetics of total CD4⁺ and CD8a⁺ T cell abundance in nasal mucosa and lungs (*n* = 5 mice per time point). The central line indicates the median; box limits represent the interquartile range (IQR); whiskers extend to data points within 1.5× IQR, and points beyond this range are shown as outliers. **C** Heatmap showing log₂ fold change in the abundance of T cell subtypes in nasal tissue, lungs, and draining lymph nodes post immunization (*n* = 5). Fold changes were calculated by dividing values of OMV vaccinated samples by PBS controls, log₂-transformed, and capped at −1 and +1 to enhance visualization. **D** Heatmap showing changes in expression of key activation markers by T cell subtypes across nasal tissue, lungs, spleen, and lymph nodes (*n* = 5). Values are log₂-transformed and capped at −4 and +4 for clarity. Pairwise comparisons between PBS and OMV groups were performed using a t-test or Wilcoxon rank-sum test, following Shapiro-Wilk normality testing (*n* = 5 mice per group). Significance stars indicate *p*-values as follows: * *p* ≤ 0.05; ** *p* ≤ 0.01; *** *p* ≤ 0.001; ns, not significant.

To delineate adaptive immune activation following innate priming by OMV-based vaccine candidates, we analyzed the kinetics of T cell subsets in the nasal tissue, lungs, draining lymph nodes, and spleen using spectral flow cytometry. Following OMV immunization, a rapid accumulation of T cells was observed in the nasal tissue as early as 6 h post-immunization. Both CD4⁺ and CD8⁺ T cell compartments contributed to this influx, with CD8⁺ T cells displaying a slightly earlier enrichment. In contrast, both subsets transiently decreased in the lungs before rebounding between 48 h and 72 h (Fig. 5A, B).

To study functional differentiation, T cells were stratified into naive (CD44⁻CD62L⁺), central memory (TCM: CD44⁺CD62L⁺), and effector memory (TEM: CD44⁺CD62L⁻) phenotypes (Fig. 5C, Supplementary fig. 8). In the nasal tissue, TCM cells showed an early and robust increase in both CD4^+^ and CD8a^+^ compartments (CD4: ~5% absolute increase; *p* = 0.002; CD8: ~33% absolute increase; *p* < 0.0005) in immunised mice relative to PBS controls. TEM cells expanded later, with significant increases detected at 72 h (CD4: ~11% absolute increase; *p* = 0.03; CD8: 27% absolute increase; p < 0.0005) in immunised mice in comparison to control ones (Fig. 5C, Supplementary fig. 8). By 72 h, both CD4⁺ and CD8^+^ TEM cells also increased in the lungs (CD4 TEM: 4% absolute increase; *p* = 0.07, CD8 TEM: 4% absolute increase; *p* = 0.05), spleen (CD4 TEM: 6% absolute increase; *p* = 0.005, CD8 TEM: 5% absolute increase, *p* = 0.003), and lymph nodes (CD4 TEM: 3% absolute increase; *p* = 0.005, CD8 TEM: 2% absolute increase, *p* = 0.03) (Fig. 5C, Supplementary fig. 8). These patterns indicate either local differentiation or systemic redistribution of memory T cells across mucosal and systemic compartments following intranasal OMV immunization.

Phenotypic profiling of activation markers supported these findings. CD4⁺ T cells in the nasal tissue upregulated CD5, CD25, and CD103 at early time points (6–24 h), with log_2_ fold changes of ~0.6–1.0 relative to PBS controls. In contrast, CD8⁺ T cells showed only modest and transient increases in CD5 and CD25 (Fig. 5D).

### Tissue-specific interactions between immune cells and OMV-based vaccine particles following intranasal delivery

**Fig. 6:**
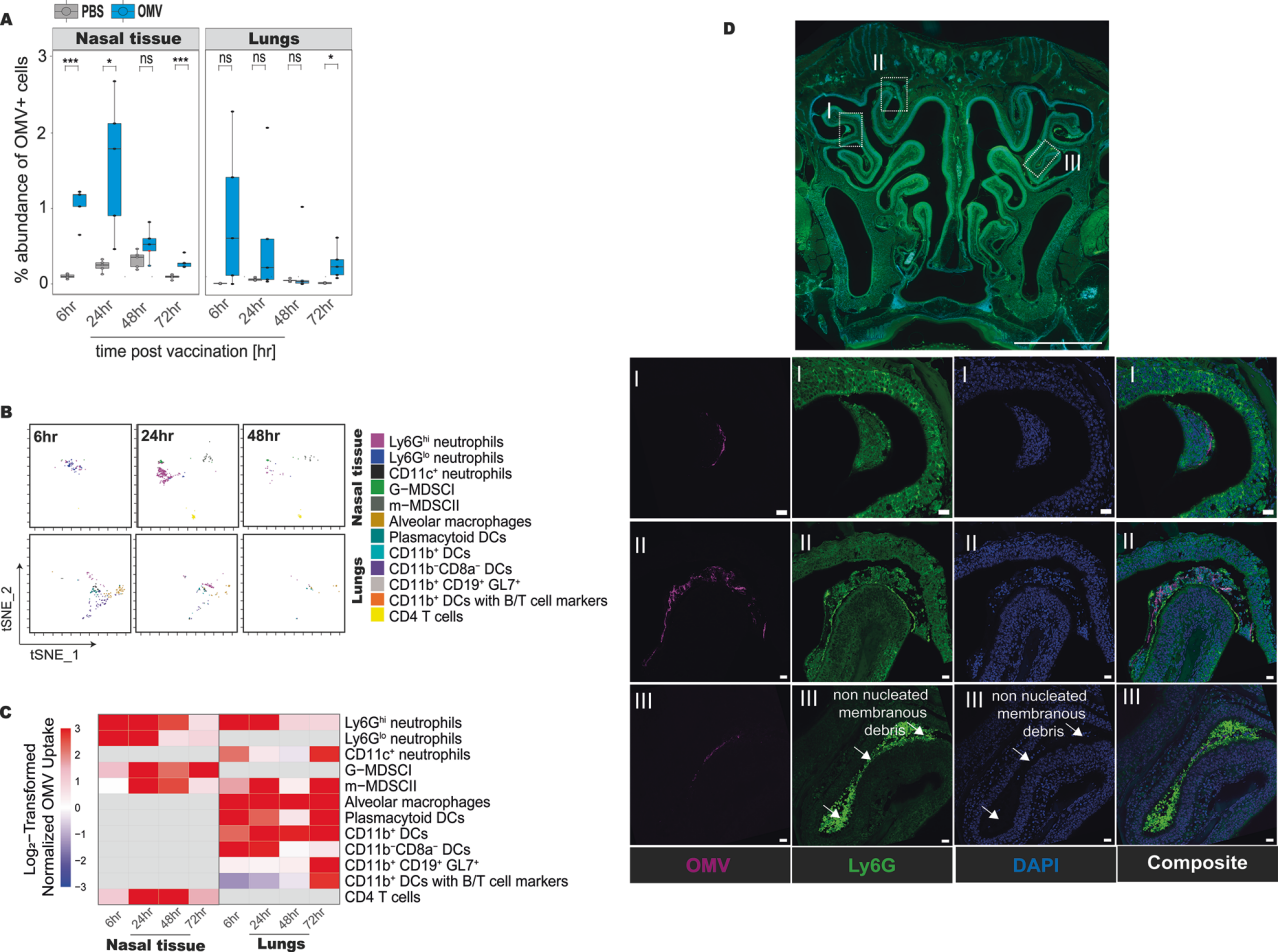
Tissue-specific immune cell localization and uptake of OMV-based vaccine particles following intranasal delivery. **A** Box plots depicting the percentage abundance of OMV positive cells in nasal tissue and lungs as determined by flow cytometry. Pairwise comparisons between PBS and OMV groups were performed using a *t*-test or Wilcoxon rank-sum test, following Shapiro-Wilk normality testing (*n* = 4–5 mice per group). Significance stars indicate *p*-values as follows: * *p* ≤ 0.05; ** *p* ≤ 0.01; *** *p* ≤ 0.001; ns, not significant. **B** tSNE plots showing distinct immune phenotypes involved in the uptake of OMV-based vaccine particles in the murine nasal tissue and lungs from 6 h to 48 h post immunization. Cells were gated on DyLight650⁺ CD45⁺ live events from nasal tissue and lung and clustering analysis (FlowSOM) was performed on the cells belonging to that specific gate. Clusters correspond to known innate and lymphoid lineages exhibiting OMV-associated fluorescence intensity as described in the legend on the right side of the tsNE-plots (*n* = 5). **C** Heatmap showing normalized mean abundance of OMV⁺ cell populations. Abundance values from OMV-vaccinated samples were normalized based on non-specific background detected in PBS controls; a factor of 1 was added to prevent division by zero. Data were log₂-transformed and values capped between −3 and +3 to enhance visualization (*n* = 5). **D** Confocal fluorescence microscopy confirming the presence and colocalization of OMV particles and neutrophils in the murine nasal cavity 6 h post intranasal immunization. Top: overview cross-section of the nasal cavity (AI sample finder, 5× objective). Insets I–III: high-resolution tile scans (LSM900, 63×) showing Ly6G⁺ neutrophils (green), DyLight650-labelled OMVs (magenta), and DAPI-stained nuclei (blue). Scale bars: overview 0.1 mm; insets I–III, 10 μm, *n* = nasal tissue sections from 3 independent mice.

To identify immune cell populations directly interacting with OMV-based vaccine particles post intranasal administration, we performed unsupervised clustering using FlowSOM on DyLight650⁺ gated CD45⁺ live cells from murine nasal tissue and lungs (Fig. 6A). This analysis revealed distinct clusters corresponding to known immune lineages, each showing evidence of OMV uptake based on fluorescence intensity (Supplementary fig. 9).

In the nasal tissue, OMV-associated fluorescence was predominantly localized to Ly6G^hi^ neutrophils, which showed the highest normalized enrichment, reaching up to 6.07-fold (log_2_) above background i.e., samples from PBS treated animals (*p* = 0.02). MDSCs and a subset of CD4⁺ T cells also demonstrated OMV interaction with maximum enrichment of ~3.82-fold (log_2_) (*p* = 0.014) and 2.5-fold (log_2_) (*p* = 0.02), respectively, indicating rapid engagement of both innate and adaptive compartments in the mucosal tissue (Fig. 6B, C).

In contrast, the lung environment displayed a broader spectrum of OMV-engaged immune cell subsets. Alveolar macrophages and plasmacytoid dendritic cells demonstrated the most robust uptake, with enrichment up to 6.81-fold (log_2_) (*p* = 0.021) and 6.75-fold (log_2_) (*p* = 0.02), while CD11b⁺ dendritic cells reached up to 6.21-fold (log_2_) (*p* = 0.07) relative to background (PBS controls). In addition, OMV-associated fluorescence was detected in specific CD11b^+^ DCs co-expressing markers of T and B lymphocytes (Fig. 6B, C). In contrast to respiratory tissues, vaccine positive cells were not observed in either lymph nodes or spleen.

Among all analyzed populations, Ly6G^hi^ neutrophils emerged as the dominant OMV-engaged cell type in the nasal tissue. This finding was further validated by confocal microscopy of murine nasal cavity sections at 6 h post-immunization. High-resolution optical sections of nasal turbinates and nasal passages confirmed the presence and colocalization of OMV particles with Ly6G⁺ neutrophils, underscoring their central role in early vaccine capture (Fig. 6DI, II). Notably, we also observed brightly fluorescent, non-nucleated Ly6G⁺ structures adjacent to intact immune cells and OMV particles consistent with cellular debris (Fig. 6DIII). These structures likely represent products of OMV-induced neutrophil activation and in situ NETosis.

Together, these results highlight the tissue- and subset-specific engagement of both innate and adaptive immune cells with OMV-based vaccine particles, illustrating distinct patterns of vaccine particle interactions in the upper and lower respiratory tract.

### CLEM-FIB/SEM reveals spatial approximation of OMV vaccine signal within neutrophils in the nasal tissue

**Fig. 7:**
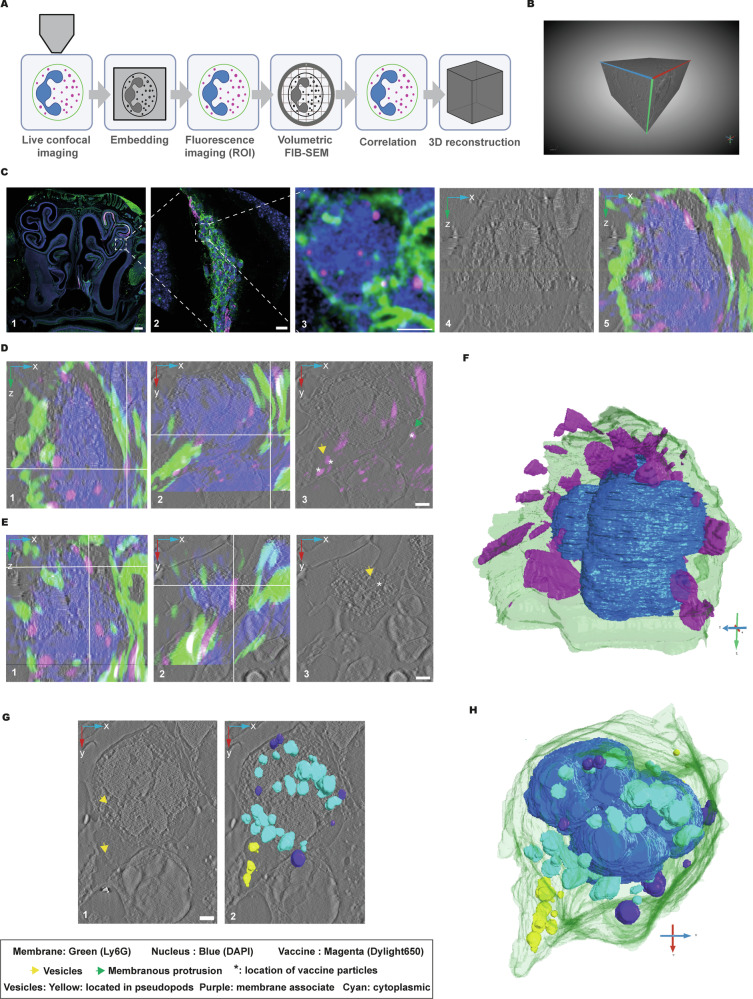
Correlative fluorescence and volumetric FIB-SEM imaging reveals subcellular interactions of OMV-based vaccine particles with neutrophils in the nasal tissue. **A** Schematic of the workflow: Region of interests (ROI) for CLEM workflow were identified using confocal imaging (LSM 900 upright, 5x and 40x objectives) on vibratome-sectioned (100 µm) immunostained nasal tissue collected at 24 h after intranasal immunization of mice with the OMV-based vaccine candidate. After ROI excision, samples were high-pressure frozen, embedded in resin and reimaged (40x tile scan; LSM 900 upright) to confirm cell identification. The selected cells were imaged using volumetric FIB/SEM at 5 nm voxel resolution, followed by correlation to the confocal images by manually registering the landmarks (BigWarp; FIJI) from stained membrane (Ly6G, green) and nucleus (DAPI, blue) of the cell of interest with the corresponding FIB/SEM slices. Finally, the nucleus, membrane, and vesicles of the cells were segmented to reconstruct the cellular model in three dimensions (3D). **B** 3D view of the volumetric FIB/SEM block showing the ROI selected for ultrastructural analysis. **C** Multiscale fluorescence and EM correlation: (1) overview of nasal cavity section (LSM 900 upright, 5x, scale bar: 0.1 mm); (2) ROI showing colocalization of OMV particles with Ly6G⁺ neutrophils (LSM 900 upright, 40x water immersion objective, scale bar: 10 μm); (3) confocal detail of the target cell imaged post embedding (LSM 900 upright, 40x water immersion objective, scale bar: 10 μm); (4–5) aligned FIB/SEM slices correlated with confocal landmarks (scale bars: 1 μm). **D** Representative orthogonal FIB/SEM views showing OMV signal (magenta) adjacent to the plasma membrane (green) and within pseudopod-like projections (D3: asterisk denotes the location of OMVs, yellow arrow points towards the vesicle, green arrow denotes the membranous protrusion found in the vicinity of OMV uptake, scale bar: 1 μm). **E** Representative orthogonal view of FIB/SEM slices highlighting OMV proximity to intracellular vesicles (yellow arrows) (scale bar: 1 μm). **F** 3D rendering of the neutrophil showing nucleus (blue), plasma membrane (green), and correlated OMV presence (magenta). **G** Representative FIB/SEM slice indicating the presence of pseudopodal extensions and vesicles localized in the pseudopods and cytoplasm of neutrophil (yellow arrow) (1) a corresponding overlay of 3D reconstructed vesicles on the same FIB/SEM slice (pseudopod associated: yellow, membrane associated: purple, cytoplasmic: cyan) (scale bar: 1 μm). **H** 3D rendering of the neutrophil illustrating the spatial presence and distribution of vesicles within the ultrastructural context. Scale bars: 0.1 mm (C1); 10 μm (C2); 10 μm (C3); 1um (C4–H). Grap**h**ic (A) generated by using BioRender.

To gain ultrastructural insight into how OMVs interact with neutrophils in the nasal tissue, we used a correlative light and electron microscopy (CLEM) workflow, combining airyscan super resolution microscopy with volumetric focused ion beam scanning electron microscopy (FIB-SEM) (Fig. 7A, B). A 100 μm vibratome section of nasal tissue from an intranasally OMV-immunized mouse was first stained for Ly6G to label neutrophils and imaged by airyscan super resolution microscopy to identify regions of interest (ROIs) showing vaccine particle uptake (Fig. 7C1, C2).

The ROI was excised, high pressure frozen using live μ (CryoCapCell). The frozen samples were freeze substituted in HM20 resin, and re-imaged using airyscan high resolution microscopy to re-locate the previously identified region of interest. Subsequently, a particular region confined to a cell of interest was identified (Fig. 7C3). This cell was then targeted for volumetric FIB-SEM imaging (Fig. 7C4). Afterwards, serial EM slices (Supplementary video 1) were aligned with confocal z-stacks using BigWarp (Fiji), based on manual landmark registration from the nucleus (DAPI) and plasma membrane (Ly6G) (Fig. 7C5). This process allowed us to target the spatial position of DyLight650-labeled OMV particles within the ultrastructural context of the reconstructed cell.

Using an integrated imaging workflow combining airyscan high resolution microscopy with volumetric FIB/SEM, we achieved in situ 3D ultrastructural visualization of neutrophil interactions with OMV-based vaccine particles in the nasal tissue (Video 1). The fluorescence signal corresponding to OMV particles was primarily localized to three key subcellular regions: adjacent to the plasma membrane (Fig. 7D1, D2), within pseudopodial projections (Fig. 7D2, D3), and in close proximity to intracellular vesicles (Fig. 7E1-3). A particularly interesting observation was the presence of internalized OMV particles in the perinuclear region of the neutrophil (Fig. 7E1, F). In addition, we observed the formation of membranous protrusions at sites where OMVs contacted the plasma membrane. (Fig. 7D3) Volumetric segmentation also revealed neutrophils with complex 3D morphologies, including pseudopod-like membrane projections and a vesicle-rich cytoplasm (Fig. 7G, H), indicating structural adaptation during antigen engagement.

## Discussion

Intranasal immunization elicits immune responses within the uniquely controlled mucosal environment of the respiratory tract, where the host mounts rapid defenses against pathogens, while preserving tissue integrity^20^. In this study, we delineated the early events triggered by OMV-based vaccine candidate immunization and organized them into three interrelated immunological domains: (1) inflammation and its regulation within the nasal tissue, (2) antigen uptake and myeloid cell activation, and (3) early signatures of adaptive priming and memory imprinting. This domain-based framework enables a spatial and temporal dissection of mucosal immune induction and its coordinated progression towards systemic immunity. Importantly, the OMV-based pneumococcal antigen display as well as the pneumococcal antigens have previously been shown to confer protection against pneumococcal colonization in vivo^15–18,21–23^. The present work builds on this foundation by providing mechanistic insight into the early mucosal immune dynamics elicited following intranasal immunization.

The nasal tissue is highly adept at initiating localized immune responses but remains particularly susceptible to tissue damage from excessive inflammation. Following intranasal administration of an OMV-based vaccine candidate, we observed a rapid and robust influx of neutrophils and classical monocytes into the nasal tissue and its draining lymphoid compartments, with recruitment peaking at 24 h. This early wave of cellular infiltration likely reflects strong local antigen sensing and Pathogen Recognition Receptor (PRR)-mediated activation, particularly Toll-like receptors, by OMV components^24^.

Phenotypic analysis revealed striking compartment-specific differences in neutrophil subsets infiltrating the upper (nasal) and lower (pulmonary) airways. The nasal tissue was dominated by highly inflammatory CD11b^hi^Ly6G^hi^ neutrophils, whereas the lungs predominantly harbored CD11b^lo^Ly6G^lo^ subsets, suggestive of distinct functional adaptations shaped by tissue-specific cues. These findings align with growing evidence for neutrophil plasticity, wherein neutrophil phenotypes are modulated by the local microenvironment^25^. Such compartmental specialization may be critical in balancing effective immune activation with the need to minimize tissue damage at mucosal sites.

The close temporal alignment between OMV presence in the nasal tissue and the peak in leukocyte infiltration at 24 h supports a model in which early innate sensing drives the recruitment and phenotypic shaping of infiltrating myeloid cells. While local chemokine or cytokine production was not directly measured in this study, engagement of pattern-recognition receptors by OMV-associated ligands is well established to initiate chemotactic programs that promote neutrophil and monocyte trafficking^3,4,26^. Future studies combining mediator profiling with single-cell phenotypic analyses will be important to more directly link inflammatory signals to the functional states observed here.

Of note, we identified the selective recruitment of phenotypically distinct myeloid-derived suppressor cells subsets to the nasal tissue, hardly present in the lungs and completely absent in secondary lymphoid tissues. Although MDSCs are typically associated with cancer or chronic inflammation^27,28^, their presence following intranasal immunization suggests a potential regulatory role, possibly limiting neutrophil-driven tissue injury while preserving immunological homeostasis. This observation raises the possibility of a novel immunoregulatory axis at mucosal surfaces, involving MDSC interactions with neutrophils, dendritic cells, or T cells during the initial stages of immune activation.

Previous studies on intranasal subunit immunization using soluble antigens, with or without adjuvants, have consistently reported rapid innate immune activation in the upper airways, often dominated by transient neutrophil and monocyte recruitment^29^ followed by relatively short-lived antigen presence in the nasal compartment^30^. In contrast, particulate delivery strategies including viral vectors, nanoparticles, and vesicle-based platforms are designed to shape early mucosal immune engagement by influencing antigen distribution, persistence, and cellular uptake^31^. In this context, our multimodal analysis shows that OMV delivery leads to nasal antigen retention, thereby driving strong neutrophil-centered mucosal immune activation alongside the emergence of MDSC-like populations. While direct comparison with alternative formulations was not performed here, the immune dynamics observed are consistent with features attributed to intrinsically adjuvanted particulate platforms and extend existing models of early mucosal immune activation by adding spatial and cellular resolution.

For vaccines to elicit robust immune response, their antigens must be efficiently internalized by antigen-presenting cells (APCs) and processed in a way that promotes immunogenicity rather than tolerance^32^. Our biodistribution data revealed that OMV particles persisted in the nasal tissue for up to 48 h and were detectable in the lower respiratory tract within hours following administration. This local presence likely provided a temporal window sufficient for antigen sampling by both resident and infiltrating myeloid cells.

Flow cytometry analysis revealed Ly6G^hi^ neutrophils and MDSC-like populations to be major players in antigen uptake in the nasal tissue. In corroboration with our data, emerging evidence indicates that MDSC-like populations are capable of antigen uptake and may influence downstream antigen presentation pathways^33^. Furthermore, flow cytometry analysis revealed a distinct vaccine engagement signature of lungs, characterized by uptake within classical antigen-presenting cells, including alveolar macrophages, pulmonary dendritic cells, and plasmacytoid dendritic cells. These subsets are well recognized for their tissue-specific roles in antigen presentation and cytokine-mediated immune modulation, particularly in the context of respiratory immunity^34–36^.

Functionally, myeloid cells displayed strong and transient upregulation of CD80, CD86, MHCII, and CD103 post-immunization, especially in the nasal tissue. This activation profile reflects the intrinsic adjuvanticity of the OMV platform, which has been shown to engage pattern recognition receptors and induce potent APC activation^3,5^. A notable highlight was the upregulation of MHCII in Ly6G^hi^ neutrophils, observed in the nasal tissue, further strengthening the hypothesis that these cells were directly exposed to and engaged with vaccine particles. This aligns with accumulating evidence that neutrophils, under inflammatory conditions, can acquire non-canonical antigen-presenting functions through MHCII expression^37^. In vivo studies have further demonstrated that neutrophils are capable of cross-presenting antigen and directly priming CD8⁺ T cells^38^, a concept particularly relevant here given the observed early expansion of CD8⁺ T cells within 6 h post-immunization.

Using a multimodal imaging pipeline integrating volumetric FIB-SEM with super resolution light microscopy, we obtained the first in situ ultrastructural reconstruction of neutrophils interacting with OMVs. These cells exhibited morphological features indicative of active engagement, including the extension of pseudopodia and membranous protrusions near OMV-associated fluorescence^39–42^. Furthermore, this also challenges the traditional view of neutrophils as passive first responders and positions them as active participants in early antigen handling.

CD103, typically linked to tissue-resident memory T cell formation^43^, and migratory APCs^44^, was unexpectedly induced across multiple myeloid subsets, including neutrophils and MDSCs. This represents a novel observation in the context of intranasal immunization and suggests the emergence of a previously underappreciated mucosal activation phenotype. Although these results point to a localized activation profile that may support T cell activation or migration to inductive sites^7^, the precise functional consequences remain to be explored.

The induction of durable T cell responses remains a hallmark of effective immunization, contributing critically to long-term immune protection^45,46^. In our model, both CD8⁺ and CD4⁺ T cells exhibited rapid activation within the nasal tissue, as evidenced by the upregulation of early activation markers CD5 and CD25 within hours after OMV administration. These early activation events coincided with the emergence of central memory (TCM) T cell subsets. This early shift towards a central memory phenotype in nasal tissue could be a result of bystander activation, a process in which T cells are indirectly stimulated in response to cytokine release from activated myeloid cells^47–49^. Similar mechanisms have been described in pulmonary settings, where cytokine-rich microenvironments amplify neutrophil recruitment and indirectly influence lymphocyte dynamics^47^.

The prominent shift toward effector memory phenotype (TEM) at later time points (72 h), particularly among CD8⁺ T cells in the nasal tissue, suggests the establishment of local memory imprinting, a process likely driven by antigen presentation within the mucosal tissue microenvironment. Although such imprinting is well described in the setting of respiratory infections^50^, our findings indicate that intranasal subunit immunization may similarly initiate tissue-specific memory programming.

Interestingly, the T cell response in the lungs displayed a temporal delay relative to that in the nasal tissue. This asynchronous pattern may reflect differences in the local APC landscape or the influence of regulatory signals originating from monocytes and other early responders^51^. Nevertheless, the expansion of TEM cells at 72 h post-immunization in respiratory mucosa as well as lymphoid tissues point toward both local as well as systemic T cell engagement post OMV immunization.

Although this study provides a detailed characterization of early immune dynamics following intranasal OMV-based immunization, a few limitations should be acknowledged. First, as our focus was on delineating the early spatiotemporal dynamics of innate and adaptive immune activation following intranasal OMV-based immunization, we did not assess antigen specificity, cytokine production, or tissue-resident status of activated T cells, nor did we include non-conventional T cell subsets such as MAIT^52^ and γδ T^53^ cells, which are known to respond rapidly to mucosal immune stimulation. These aspects, which are critical for understanding long-term protective immunity and early innate-adaptive crosstalk, warrant dedicated investigation in future studies using approaches such as tetramer staining, cytokine profiling, residence markers (e.g. CD69), and inclusion of appropriate lineage-specific markers. Second, distinguishing monocytic MDSCs from activated macrophage populations remains challenging due to overlapping surface phenotypes in the context of an inflammatory environment. Expanding the myeloid panel with additional markers such as Siglec F^54,55^ or MerTK may help to improve resolution of these subsets. Third, we did not detect early drainage of vaccine particles to the draining lymph nodes, which may reflect limited antigen transport or delayed presentation following intranasal administration, as reported in previous intranasal immunization studies^54,55^. In addition, intranasal delivery as a liquid bolus is expected to favor retention within the upper airways and does not recapitulate aerosolized deposition into the distal lung (e.g., 1–3 μm particles)^56^, which should be considered when interpreting biodistribution patterns in the lower respiratory tract. Together, these route- and formulation-dependent factors likely shape early antigen distribution and downstream immune engagement following intranasal immunization. Despite these limitations, our findings outline a layered and spatially coordinated immune response, rapid but regulated inflammation in the nasal tissue, antigen capture involving both classical and unconventional myeloid subsets, and early T cell priming within mucosal tissues preceding systemic dissemination. The prominent roles of neutrophils and MDSC-like cells in antigen engagement and immunoregulation point to a more complex landscape of early mucosal immunity than previously appreciated. These insights not only deepen our understanding of innate-adaptive interplay in mucosal immunization but also highlight promising new cellular targets for optimizing the design of next- generation intranasal vaccines against respiratory pathogens.

## Methods

### Generation of nanoluciferase fusion antigens and surface display on OMVs

The nanoluciferase (nLuc) coupled pneumococcal antigens were cloned into vector pET-11C by GenScript (Supplementary Fig. 10, Table S2). The antigens were heterologously expressed in *E. coli* and affinity purified by using His-Tag affinity chromatography (AKTA, GE Healthcare).

OMVs used for antigen ligation were derived from *S. enterica* serovar Typhimurium SL3261 ΔtolRA ΔmsbB cells expressing HbpD(Δd1)-SpyCatcher, following a previously described protocol with modifications^17^. For the generation of nLuc-ligated OMVs, a proprietary production process was employed that did not alter OMV quality. Briefly, bacteria were cultured in a single-use bioreactor, and OMVs were harvested by filtration.

OMVs were incubated with purified antigen-nLuc fusion protein for 24 h at 4 °C to facilitate covalent coupling via the SpyCatcher/SpyTag interaction. Following ligation, OMVs were washed, concentrated, and stored in PBS (pH 7.4) supplemented with 15% glycerol. Antigen incorporation was verified by SDS-PAGE and Coomassie staining.

Total protein content of the OMV preparations was quantified using the DC™ Protein Assay Kit II (Bio-Rad, REF 5000112) in the presence of 4% SDS.

### Fluorescent labelling of vaccine candidate particles

To visualize the uptake of vaccine particles by microscopy as well as to analyze the uptake of vaccine particles by immune cells via flow cytometry, outer membrane vesicles (OMV) displaying the nLuc fused antigens on the surface were labelled with a fluorescent dye Dylight650 (Thermo Scientific) as per manufacturer’s instructions. Briefly, vaccine candidate particles were incubated with Dylight650 for 1 h at room temperature (RT) in the dark. The unbound dye was removed by washing twice for 2 h with PBS (Gibco) via centrifugation (25,000 x *g*, 4 °C). After the final wash, the supernatant was removed, and vaccine particles were resuspended in PBS/15% glycerol, aliquoted and stored at −20 °C for further use.

### In vivo immunization and imaging experiment

Mice experiments performed for this study were approved by the Radboudumc Committee for Animal Ethics (License no. AVD10300202216324) and were conducted in accordance with the Dutch legislation.

For all in vivo experiments, 7-week-old female C57Bl/6 J mice were purchased from Charles River, Germany. Upon arrival, mice were randomized, using Random.org sequence generator, and grouped in individually ventilated Greenline cages (IVC), a maximum of 6 mice per cage. Mice were further acclimatized for at least a week before the onset of an experiment. Allocation of mice to control and experimental groups were also done randomly. Sterile water and food were provided *ad libitum*, with daily welfare monitoring.

Mice were intranasally immunized via a 10 µl pipette with 3 µg/µl of vaccine candidate formulation in a total volume of 5 µL. The vaccine candidate was administered to both the nostrils under inhalation anesthesia. An equal volume of PBS/15% glycerol was intranasally administered to the control mice. Previously published in vivo efficacy studies^17^ served as a reference to define the OMV dosing range; however, dosing in those studies was expressed in optical density (OD) units. These studies were therefore used as a guiding reference to define an appropriate dosing range. For the present work, OMV doses were standardized to microgram quantities, and a final dose of 15 µg was selected to ensure sufficient sensitivity for in vivo bioluminescence imaging. This dose was well tolerated in mice during the observation period

In line with our previous experience with intranasal OMV formulations, mice were closely monitored for body weight and behavior following immunization. Transient weight loss was occasionally observed during the first days after administration; however, animals generally recovered within three days. No sustained adverse effects were noted during the monitoring period, and predefined humane endpoints were applied throughout the study. For in vivo bioluminescence imaging, 5 µg of furimazine (0.25 mg/kg) (Promega) in a volume of 10 µl was administered intranasally to both the nostrils of the mice (under inhalation anesthesia) at specific timepoints i.e., 1, 6, 24, 48, and 72 h post immunization. After three to five minutes of substrate administration, animals were placed in IVIS chamber under inhalation anesthesia (1.5–2.5%), and luminescence was measured. The settings for signal acquisition via the software were adjusted to preference: Luminescence, autoexposure, Binning 4-8, f/1 aperture. The acquired signals were quantified as average radiance (photons/sec/cm2/sr) by using Living Image software (version 4.3).

### Tissue harvesting and processing to single cell suspension

Post intranasal immunization, mice were dissected and perfused with 1x PBS (Gibco, pH 7.4) under inhalation anesthesia (2.5% isoflurane). Organs/tissues including lungs, nasal draining mandibular lymph nodes, spleen and nasal tissue were isolated and kept in RPMI medium on ice until further processing. Single cell suspension from nasal tissue and lungs was obtained by digesting the tissues enzymatically in the presence of Liberase (40 µg/mL, Roche) and DNase I (40 µg/mL, Roche) in 1 mL RPMI containing 0.01% Penicillin/Streptomycin (BioWest) for 30 min, at 37 ^o^C under shaking conditions. Enzymatically digested tissues i.e., nasal tissue and lungs as well as spleen and lymph nodes were further mechanically disrupted and filtered through 70 µm sieves in a total volume of 5 mL RPMI with 0.01%Pen/Strep (BioWest) and 3% FCS (Gibco). The isolated suspension was washed with a buffer containing 1x PBS (Gibco, pH 7.4), 5 mM EDTA (Thermofisher scientific) and 3% FCS via centrifugation (500 × *g*, 5 min, 4 ^o^C). Red blood cells were removed by using red blood cell lysis buffer (eBioscience). After RBC removal and washing once with the buffer (1x PBS, 5 mM EDTA and 3% FCS), the cells were finally resuspended in 1x PBS (Gibco, pH 7.4) and cell count was obtained via automated Beckman cell counter.

### Flow cytometry labelling

For antibody staining, 0.5 × 10^6^ cells from each tissue were taken in a volume of 100 µl and stained with fixable viability dye (Zombie NIR, Biolegend) diluted in PBS for 30 min at 4 ^o^C in a 96 well plate. After washing the cells with a buffer (PBS (Gibco) containing 0.2% BSA (Sigma)), the cells were incubated with 5 µl of monocyte blocker (Biolegend) and anti-mouse anti-CD16/32 (Fc blocker, Biolegend) for 10 min. Afterwards, the cells were incubated with an antibody master mix containing Brilliant Blue staining Buffer (BSB) overnight at 4 ^o^C. The optimal amount of antibodies to be used for each tissue was determined via titrations (Table S1). After overnight staining, cells were washed with the buffer (PBS/0.2% BSA) and further incubated for 30 min at 4 ^o^C with PerCP-Streptavidin to stain biotinylated Peanut agglutinin (PNA) which was added in the previous step of antibody mix. After washing the cells twice, the samples were resuspended in the buffer and data was acquired on an ID7000 5 L spectral flow cytometer (Sony). 100,000 events were included per sample. Identical protocol was used to get fluorescence minus one (FMO) control for each fluorochrome.

### Flow cytometry data analysis

After data acquisition, autofluorescence was detected in single cells of unstained sample for each tissue and added to the unmixing matrix. After autofluorescence correction, live CD45^+^ cells were gated (Supplementary figure 11) and used to check correct unmixing of fluorochromes. The unmixing matrix was adjusted wherever applicable to adjust the fluorochromes’ spectral references. Properly unmixed spectral data was exported as (parametric) fcs 3.1 files for downstream manual gating (Flowjo, 10.10) as well as for unsupervised analysis (Cytobank).

For unsupervised analysis, the online platform Cytobank was employed. The data was at first uploaded to Cytobank as fcs 3.1 files. All the fluorescent parameters were first scaled using Arcsinh and cofactor was adjusted to compress negative populations unimodally around 0. Afterwards, cells were manually cleaned for doublets, dead cells, and non-immune cells (CD45^-^). Dimensionality of high parametric data was reduced by using tSNE-CUDA algorithm^19^. Since number of immune cells varied between vaccinated and non-vaccinated mice, proportional numbers of cells were sampled for input. To identify the phenotypic distinct clusters computationally, FlowSOM was used in parallel generating 25 metaclusters and 81 clusters. For visual interpretation of the results, an overlay of tSNE plots and FlowSOM clusters was used. For both the algorithms i.e., tSNE-CUDA and FlowSOM, all fluorescent parameters other than autofluorescence and viability dye were included.

MFI cutoffs for each fluorescent marker and for each tissue were determined via FMO controls. These MFI cutoffs were used to generate a heatmap using the data generated by Cytobank via R packages (nbclust, pheatmap). These heatmaps were carefully inspected to identify key phenotypes and subtypes of immune cells.

Cluster abundances (event counts or percentages) were summed per phenotype and compared between OMV and PBS groups. In parallel, mean fluorescence intensity (MFI) values of activation markers (e.g., MHCII, CD80, CD103) were analyzed to assess expression dynamics over time. Visualizations were produced as boxplots using R packages (ggpubr, dplyr, ggplot).

To compute differences in abundance or marker expression of different immunophenotypes in different tissues across different time points, log_2_ fold change values were calculated for respective data in R. These values were then plotted into heatmaps by using R packages (pheatmap, RColorBrewer, scales). Depending upon the range of the data, the values of fold change were capped to a certain limit to enhance and ease visualization in a heatmap.

### Immunohistochemical processing and imaging

For immunohistochemical experiments, mouse heads were obtained post intranasal immunization at defined time points (6, 24, 48, 72 h). After removing the skin, ears, and whiskers, heads were fixed in EAFS fixative containing 40% ethanol, 5% acetic acid, 3.7% formaldehyde, and 45% of 0.9% sodium chloride solution for 3 days. The heads were further decalcified in 6.5% formic acid at room temperature for 4 days, after which the mouse heads were cut into 3 transversal (sagittal plane) slices, each 2 mm thick. Post 24 h of decalcification in 6.5% formic acid, 2 mm head sections were paraffinized. For immunohistochemical staining, 4–5 µm sections of paraffinized specimens were obtained via microtome. Prior to immunohistochemical staining, slides were dewaxed and rehydrated, followed by blocking with 2% bovine serum albumin in 0.05% PBS-Tween 20. The slides were individually stained for Ly6G (Biolegend, 10ug/mL). Vaccine candidate particles were labelled with Dylight650 (Thermofisher Scientific). Images were obtained by using an advanced brightfield microscope, Zeiss Axio Observer 7 with Sample AI Finder (20x) and Zeiss LSM900 (63x) with Airyscan for confocal imaging. The following channels were used in different combinations depending on the staining goal for the tissues: DAPI, AF488, AF555, and AF647.

### Tissue processing and staining for CLEM

To image the uptake of vaccine candidate particles by neutrophils/monocytes, animals were perfused under inhalation anesthesia by using 4% formaldehyde (formalin)/0.1% glutaraldehyde (5 mL) post 24 h of intranasal immunization with Dylight650 labelled OMVs. Afterwards, the head of the mice were cut. After removal of skin, eyes, and whiskers, heads were immersed in 15 mL of 4% formaldehyde/0.1% glutaraldehyde in PBS-buffer in a 50 mL falcon, overnight at room temperature. After washing once with PBS, heads were decalcified in 15 mL of EDTA for 14 days. 2 mm thick head sections behind the eyes were cut via sterile scalpel and once again decalcified in EDTA for 24 h. The sections were further cut to a thickness of 100 µm via vibratome. To quench autofluorescence, the section was incubated in a 12-well cell culture plate with PBS + 100 mM Glycine+100 mM NH_4_Cl, overnight at 4 °C. Afterwards, the head sections were blocked with 1% BSA/PBS overnight at 4 °C, followed by the addition of anti-mouse anti-Ly6G (Biolegend, 10 µg/mL) in the blocking buffer, for 16 h at 4 °C. The non-bound antibody was removed by washing with PBS 3x 1 h followed by the incubation of rabbit anti-mouse Alexafluor 568 (Thermofisher Scientific, 5 µg/mL), for 16 h at 4 °C. After subsequent washing with PBS (3x 1 h), the stained sections were kept in PBS with 0.1% PFA until final imaging.

### Fluorescent ‘live-cell’ and AFS-HM20 imaging

Fluorescent images were recorded using a LSM900 laser scanning microscope equipped with an Airyscan 2 detector (Zeiss microscopy GmbH). Prior to imaging, a Zeiss ZEN Connect project (Zeiss software for correlative microscopy, version 3.5) was created to overlay multiple low- and high-resolution images and to facilitate further correlation with other imaging modalities.

Images were obtained at room temperature in PBS using either a 5x (Epiplan-Apochromat 5x/0.2 M27, Zeiss), 10x (Epiplan-Apochromat 10x/0.4 M27, Zeiss), 40x (W Plan-Apochromat 40/1.0 M27, Zeiss, water dipping) or a 63x (W Plan-Apochromat 63/1.0 M27, Zeiss, water dipping) objective on the Airyscan Array Detector using the Multiplex SR-4Y acquisition mode. Airyscan images were further processed using the Airyscan toolbox in the ZEN software. Corresponding Reflection microscopy images were acquired using the confocal detector.

### High pressure freezing

Samples were immersed in 10% BSA (31349, Sigma) and sandwiched between HPF carriers with 2 mm internal diameter. Prior to creating the sandwich, all carriers were rinsed in pure ethanol. Samples were loaded in a 0.1 mm cavity carrier (Art. 662, Wohlwend) and a flat top (0.3 mm, Art. 242 Wohlwend). The flat side of the flat top was treated with 1% L-α-phosphatidylcholine (61755, Sigma) in ethanol (1.00983.1000, Supelco). The samples were then frozen at high pressure using live μ (CryoCapCell) and stored in liquid nitrogen until further processing.

### FIB/SEM

The sample was inserted into the Zeiss Crossbeam 550 FIB/SEM (Carl Zeiss Microscopy GmbH, Oberkochen, Germany) chamber. (The sample was tilted 54°). Regions of interest were chosen by LSM imaging. For milling coarse trenches using FIB 30 kV@30 nA probe. A protective layer of platinum was deposited on top of the region of interest. Tracking marks were milled into the Pt layer and filled with carbon to allow tracking of the FOV and autofocus/autostigmatism alignments, using the Atlas 3D software (Atlas Engine v5.3.3) to collect the 3D dataset.

Fine FIB milling on the cross section was done using the 30 kV@1.5 nA probe. For serial FIB milling the 30 kV@700pA probe current was used. After the removal of each 5 nm thick slice, the new slice surface was exposed by FIB milling, an InLens secondary and EsB image were simultaneously collected at 2.0 kV acceleration potential with 200 pA probe current. The EsB grid was set to 500 V. The milling and imaging processes were continuously repeated, and long series of images was acquired. The stack has 1284 images of 2048×1536 pixels. The voxel size of the stack was 5 × 5 × 5 nm.

### Image correlation, segmentation and 3D rendering

All processing, visualization and analysis were performed in the ORS Dragonfly software (V2024). Wherever necessary, slices of the 3D stacks were aligned using the mutual information and sum of squared differences registration method. To correlate the confocal images with the FIB/SEM slices, BigWarp plugin of FIJI was employed. Landmarks from stained membrane and nucleus from Airyscan super resolution microscopy images were identified by correlating with the corresponding FIB/SEM slices. Those landmarks were used to transform the three confocal channels individually. Transformed fluorescent channels were then overlayed with FIB/SEM stack in FIJI to generate composite images harboring the three fluorescence channels (membrane: green, nucleus: blue, vaccine candidate particles: magenta) and FIB/SEM stack. Automatic 3D segmentation was performed using deep learning-based segmentation via Arivis cloud (Zeiss), and segmentations were modified manually wherever necessary. After segmentation of membrane, nucleus and vesicles, the 3D structure of the cell was rendered in ORS Dragonfly software by converting the segmented regions into meshes. To incorporate the vaccine candidate particles in the 3D rendered cell, a binary mask from the transformed stacks of vaccine channel (AF647) (via BigWarp) was generated in FIJI and further used to generate meshes in ORS Dragonfly software with subsequent incorporation in the 3D ultrastructure of the cell.

### Statistical analysis

Statistical analyses were performed using R (v4.2.0) and GraphPad Prism (v9). For longitudinal in vivo imaging experiments involving repeated measurements from the same mice over time, repeated measures ANOVA was used to evaluate within-subject differences. In experiments where individual mice were sampled at each time point and condition, the Shapiro-Wilk test was applied to assess data normality. Depending on the distribution and variance, pairwise comparisons between PBS and OMV groups were performed using either a student’s *t*-test (for normally distributed data with equal variances), Welch’s t-test (for normally distributed data with unequal variances), or the Wilcoxon rank-sum test (for non-normally distributed data). Statistical comparisons between OMV-vaccinated and PBS control groups were performed as pairwise tests at each time point to evaluate predefined immunological parameters. Given the exploratory nature of the study and limited sample size (*n* = 5 mice per group), no correction for multiple testing was applied. Statistical significance was denoted as follows: *p* < 0.05 (*), *p* < 0.01 (**), and *p* < 0.001 (***). All tests were two-tailed, and data are presented as mean ± SEM or median with interquartile range, as indicated in the figure legends.

## Supplementary information

Supplementary Information

Supplementary video 1

## Data Availability

All data supporting the findings of this study have been deposited in the project specific data collection in Radboud Data Repository and are accessible via the following DOI: (10.34973/f82t-nt54). The dataset associated with this manuscript will be made publicly available upon publication.
