# Supplementary material for: Tracking spatio-temporal dynamics of early immune responses to an intranasal OMV-based pneumococcal vaccine candidate in mice
Source: NPJ Vaccines. 2026 Mar 30;11:105. doi: 10.1038/s41541-026-01430-y (PMC13194884; doi:10.1038/s41541-026-01430-y)
Supplement: Supplementary file 1 — Supplementary Information [file 41541_2026_1430_MOESM1_ESM.pdf]

Supplementary data

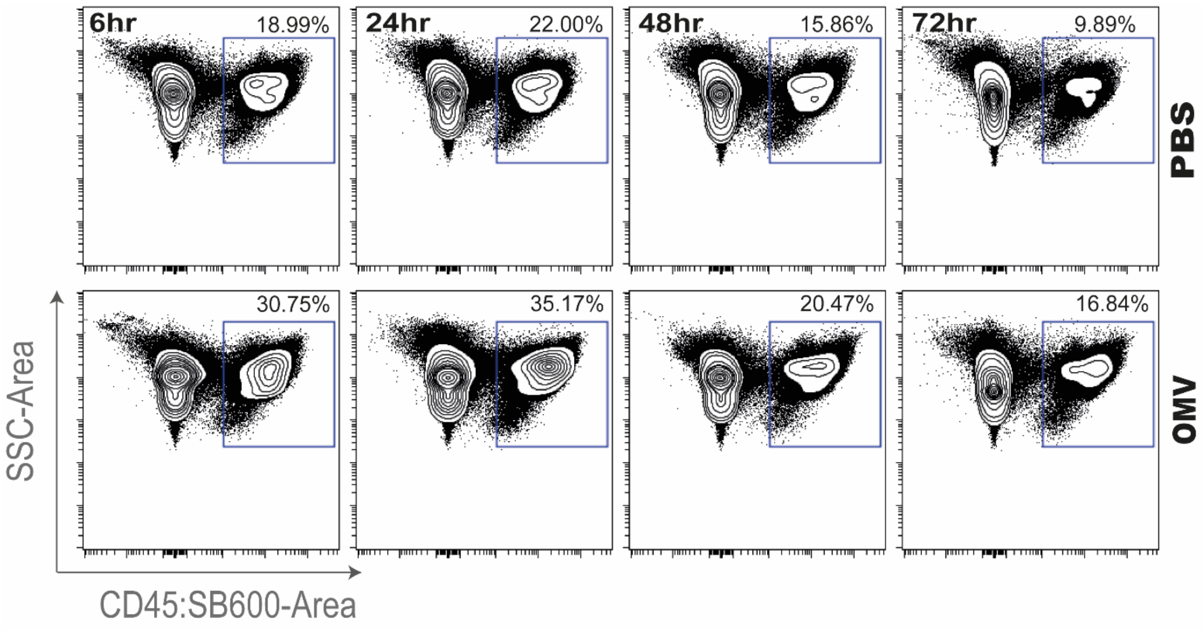

Supplementary figure 1. Contour plots illustrating the abundance of total immune cells (live CD45$^+$) in murine nasal tissue across different time points post-vaccination; plots are concatenates from individual samples, percentages represent the mean of individual samples (n = 5).

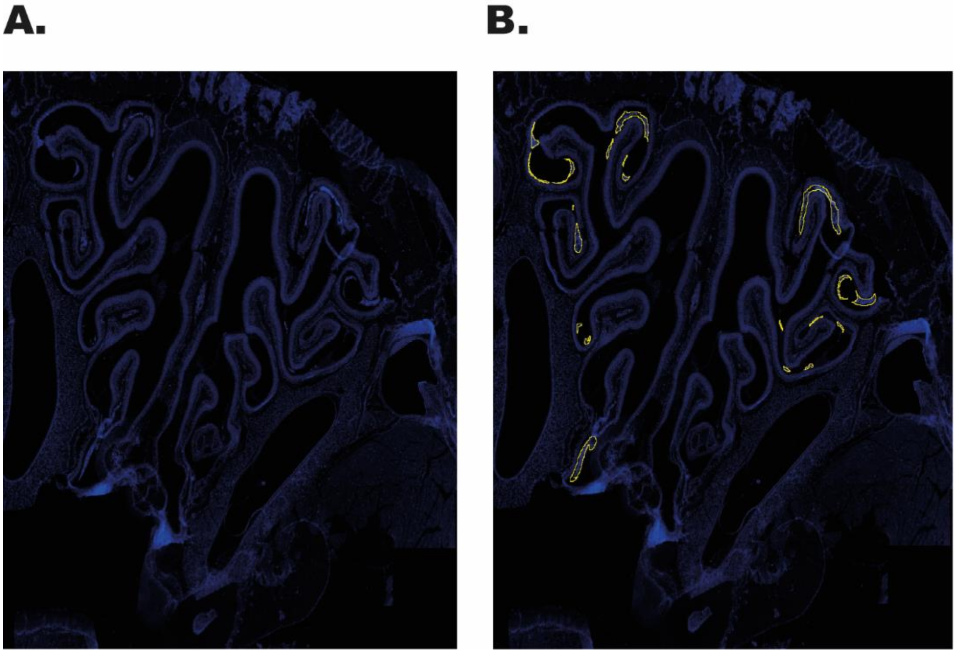

Supplementary figure 2. Method of ROI selection and quantification in nasal tissue sections. Representative image illustrating the approach for region of interest (ROI) selection and area quantification using FIJI. Regions corresponding to DAPI-positive cells within the nasal turbinates and passages were marked (yellow) as ROIs as indicated in B, and their total area was quantified to assess cellular influx.

.

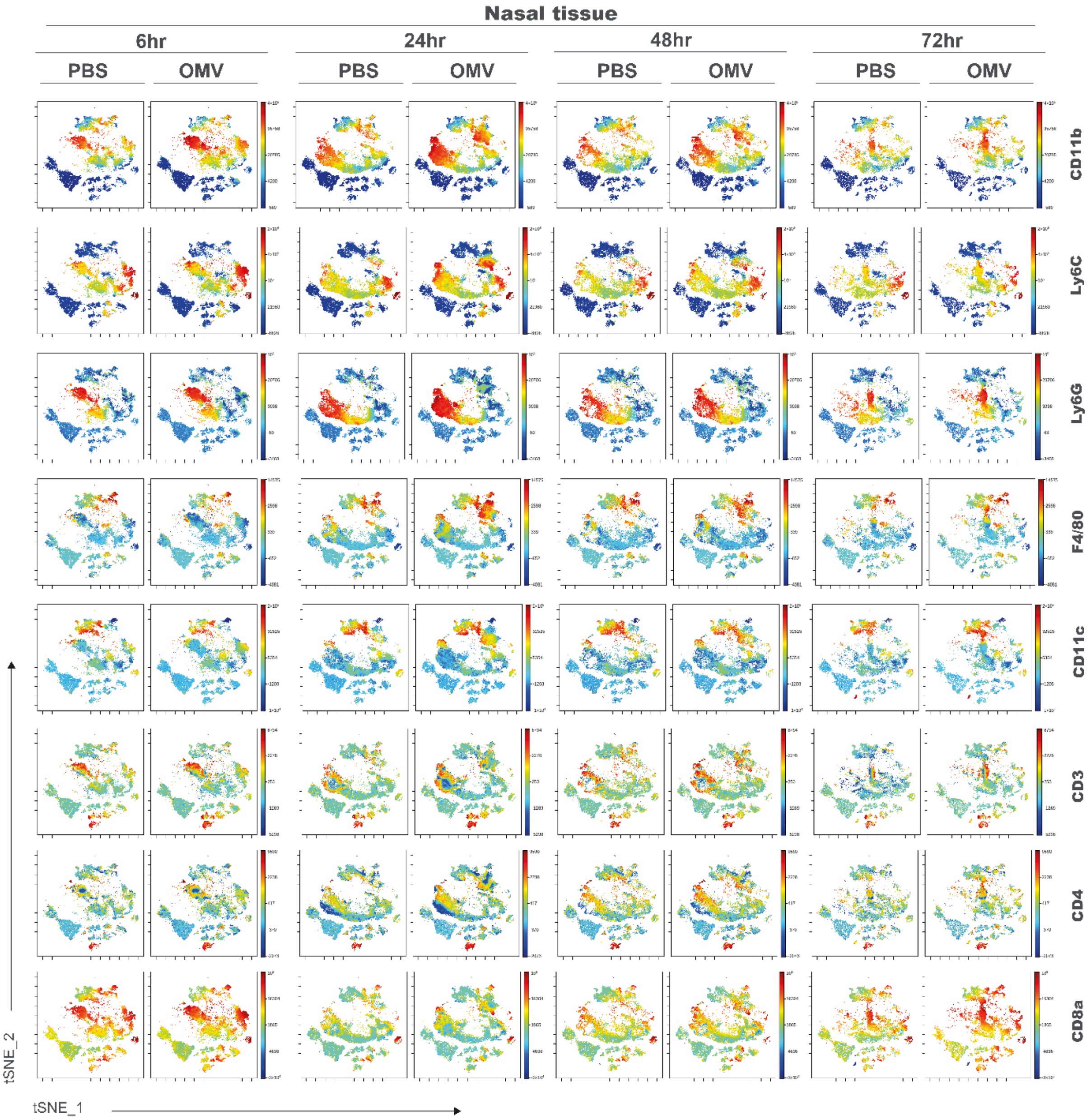

Supplementary figure 3: t-SNE plots showing marker expression patterns in nasal tissue. t-SNE plots depict the expression profiles of key markers in nasal tissue following OMV vaccination. Plots are concatenated from individual samples (n=5) for each time point and condition. Each marker is displayed with its own independent color scale to indicate relative expression intensity.

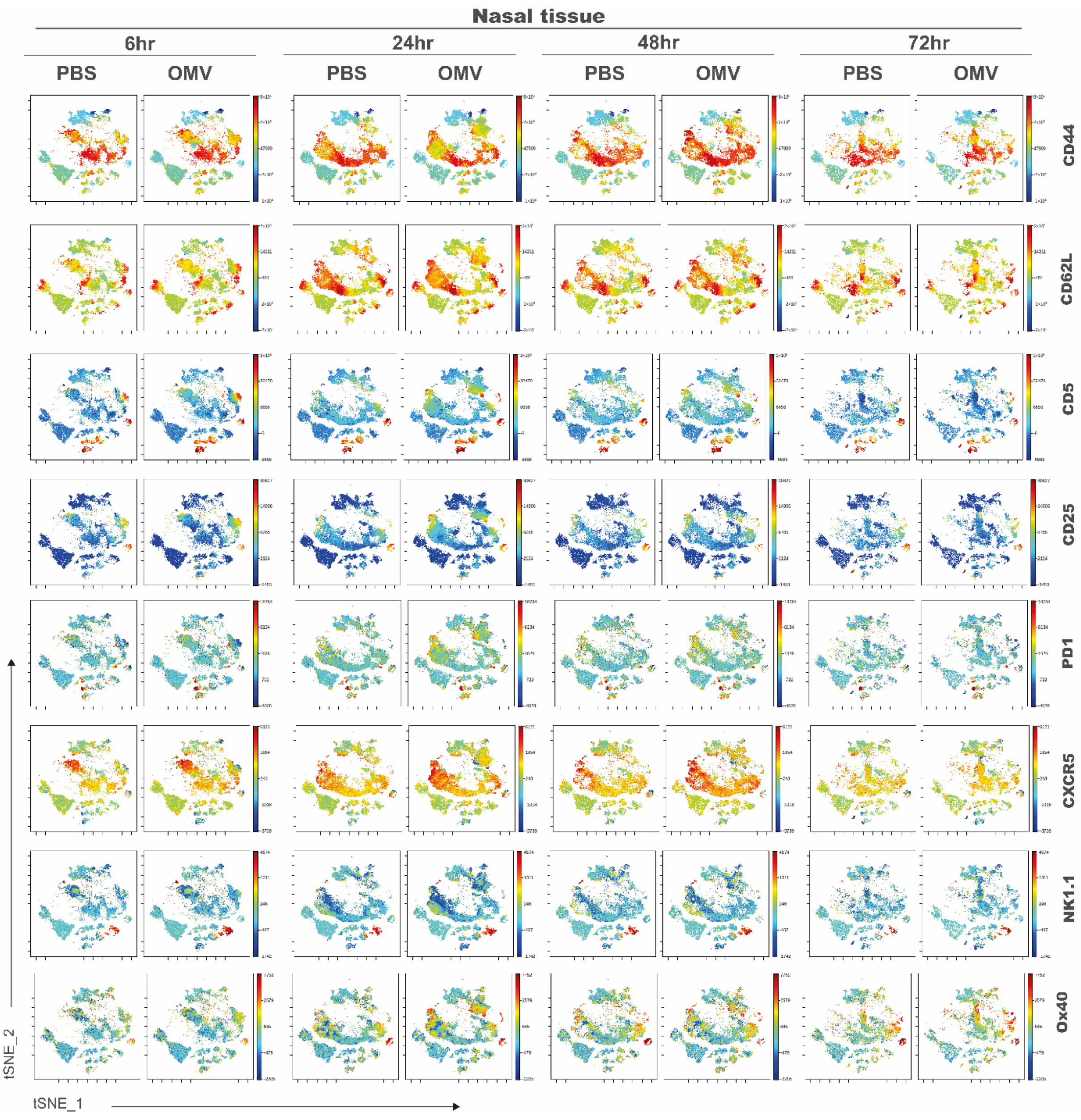

Supplementary figure 3 (continued): t-SNE plots showing marker expression patterns in nasal tissue. t-SNE plots depict the expression profiles of key markers in nasal tissue following OMV vaccination. Plots are concatenated from individual samples (n=5) for each time point and condition. Each marker is displayed with its own independent color scale to indicate relative expression intensity.

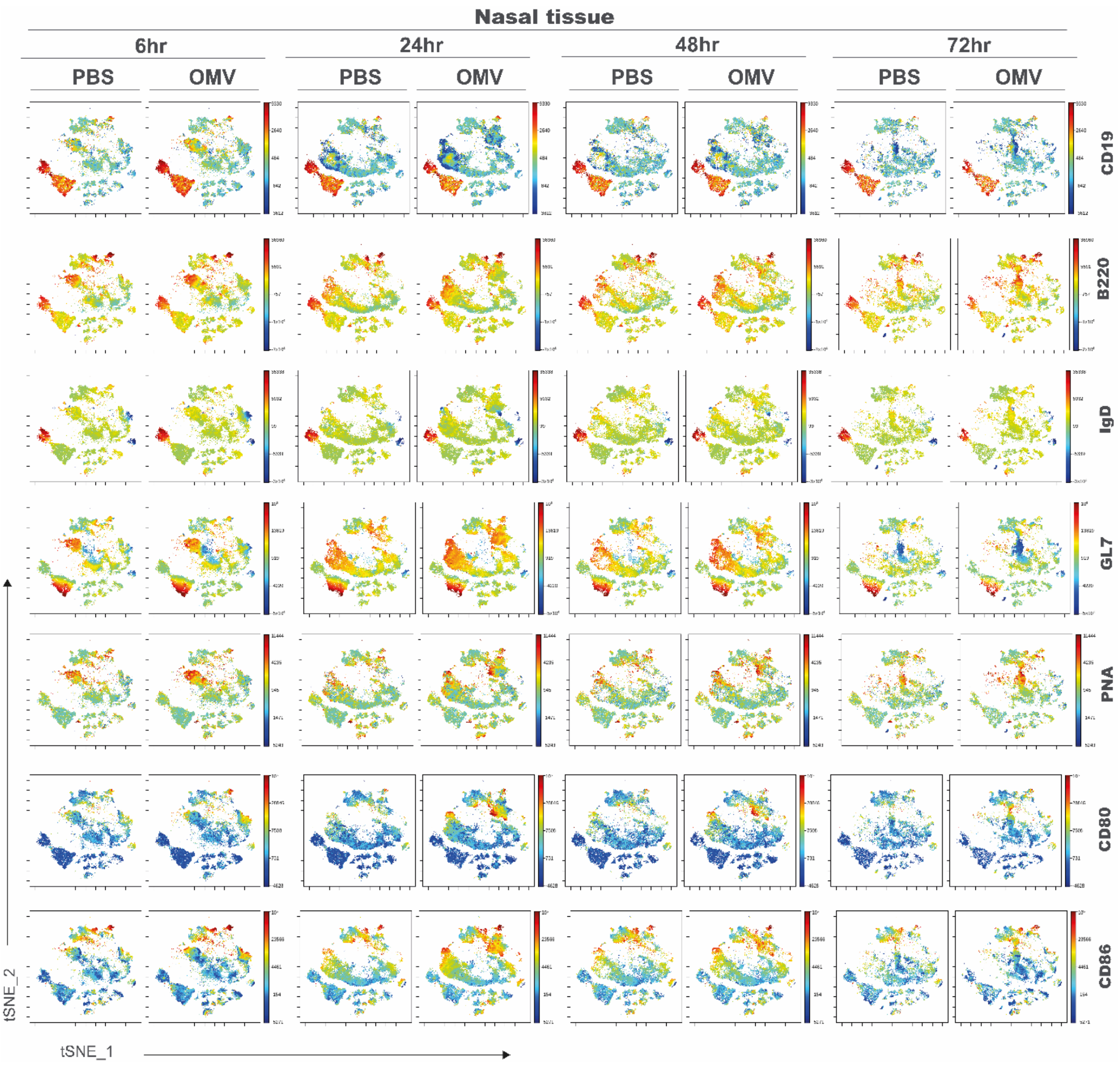

Supplementary figure 3 (continued): t-SNE plots showing marker expression patterns in nasal tissue. t-SNE plots depict the expression profiles of key markers in nasal tissue following OMV vaccination. Plots are concatenated from individual samples (n=5) for each time point and condition. Each marker is displayed with its own independent color scale to indicate relative expression intensity.

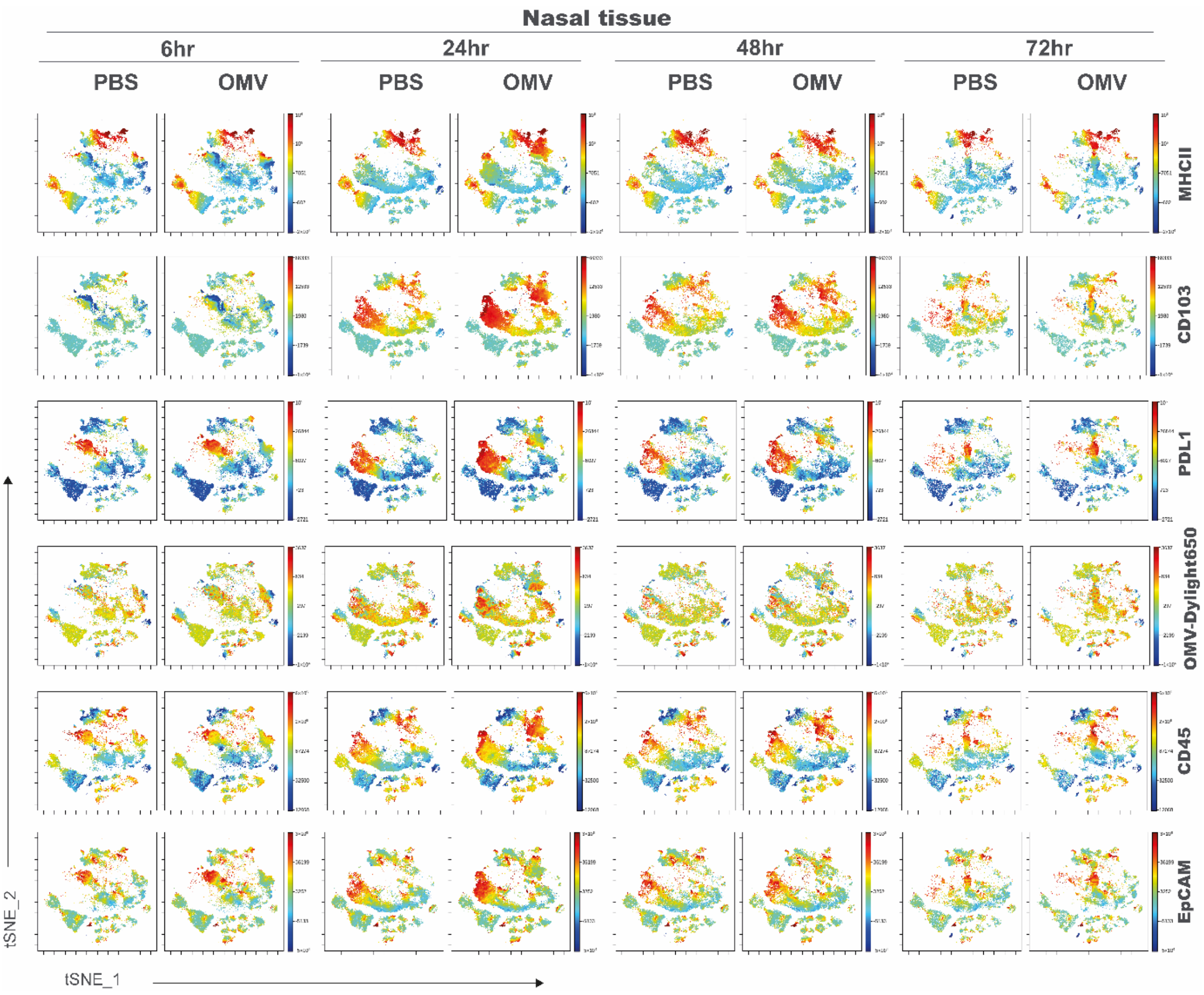

Supplementary figure 3 (continued): t-SNE plots showing marker expression patterns in nasal tissue. t-SNE plots depict the expression profiles of key markers in nasal tissue following OMV vaccination. Plots are concatenated from individual samples (n=5) for each time point and condition. Each marker is displayed with its own independent color scale to indicate relative expression intensity.

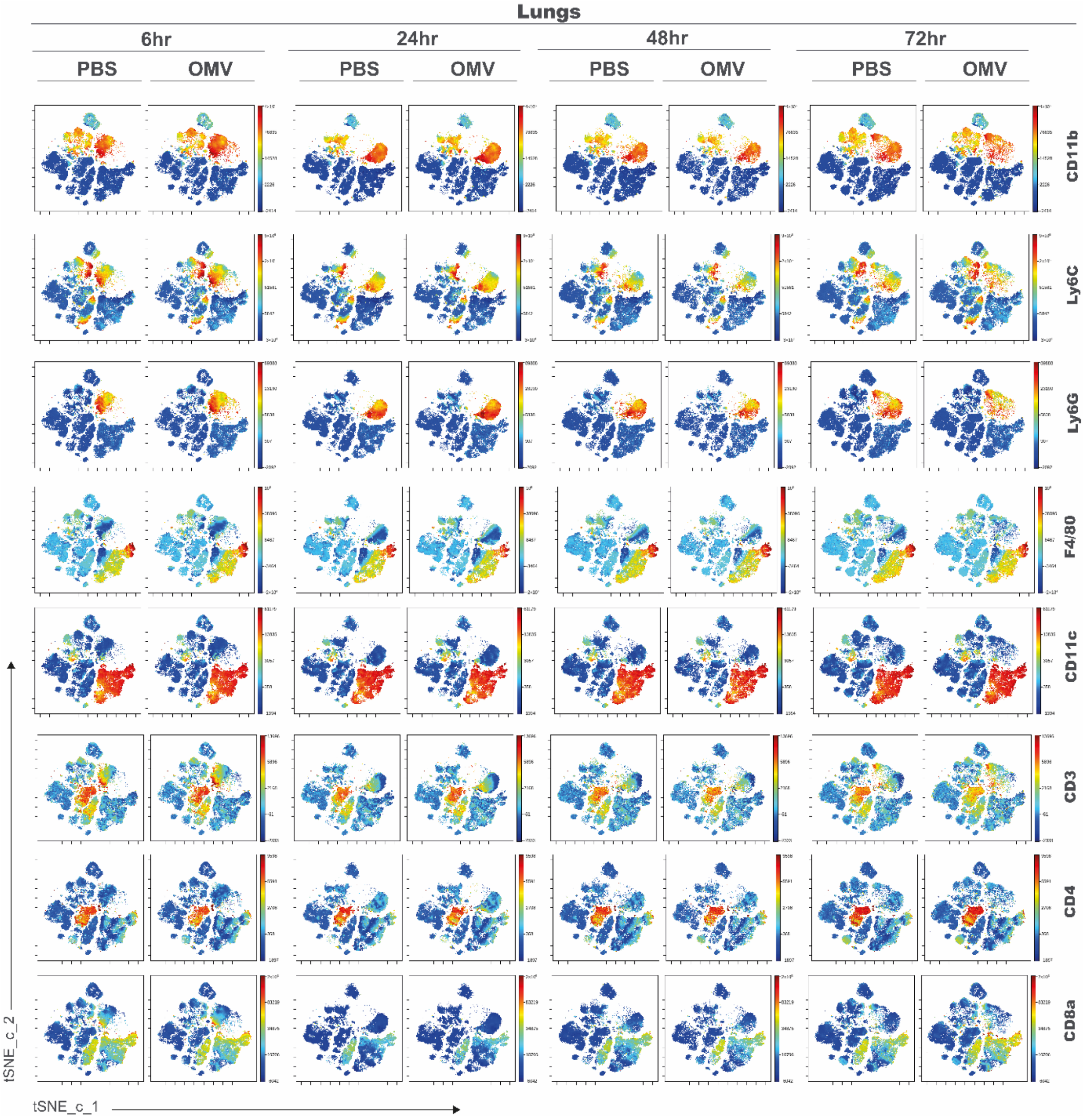

Supplementary figure 4: t-SNE plots showing marker expression patterns in lungs. t-SNE plots depict the expression profiles of key markers in lungs following OMV vaccination. Plots are concatenated from individual samples (n=5) for each time point and condition. Each marker is displayed with its own independent color scale to indicate relative expression intensity.

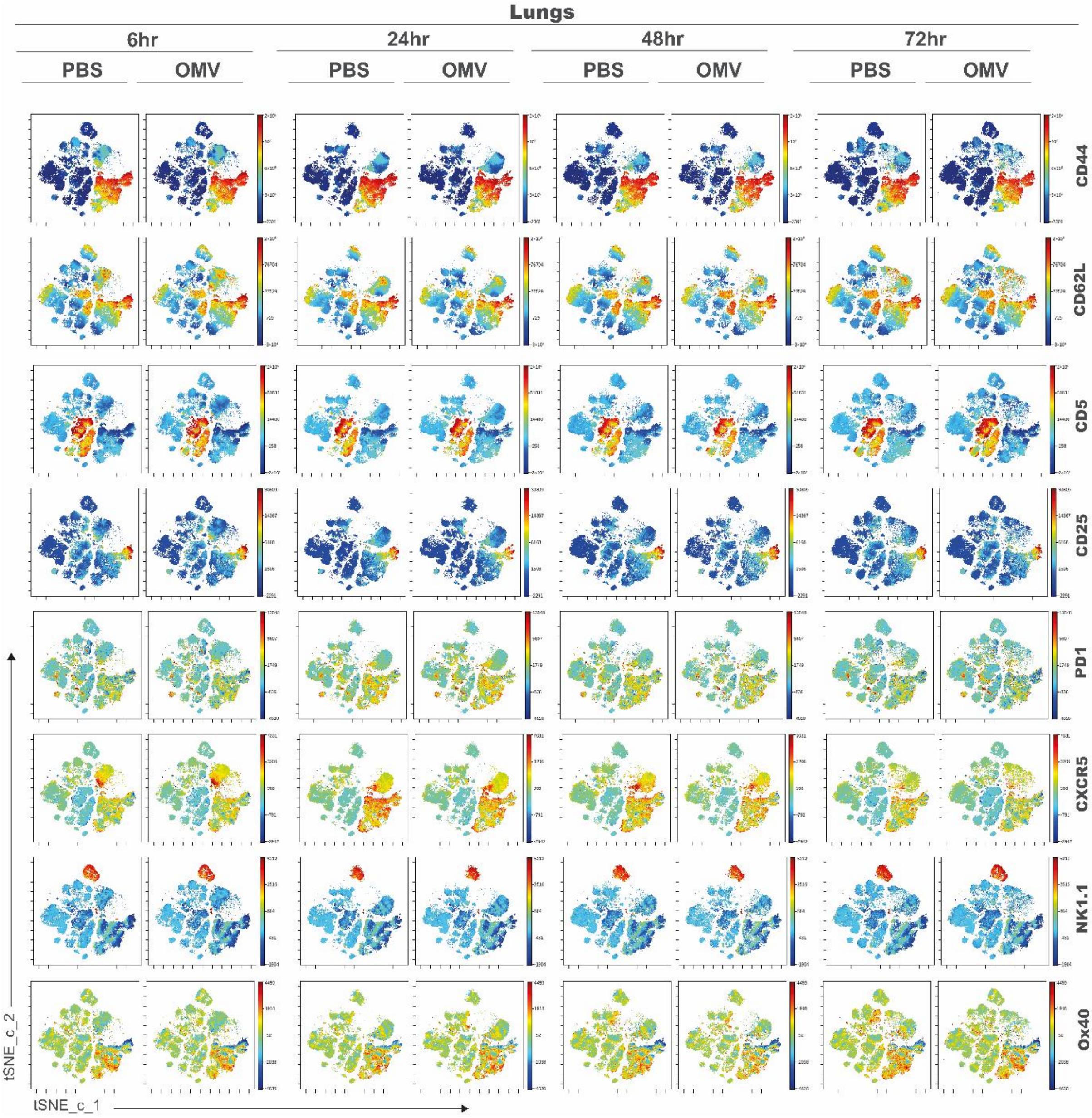

Supplementary figure 4 (continued): t-SNE plots showing marker expression patterns in lungs. t-SNE plots depict the expression profiles of key markers in lungs following OMV vaccination. Plots are concatenated from individual samples (n=5) for each time point and condition. Each marker is displayed with its own independent color scale to indicate relative expression intensity.

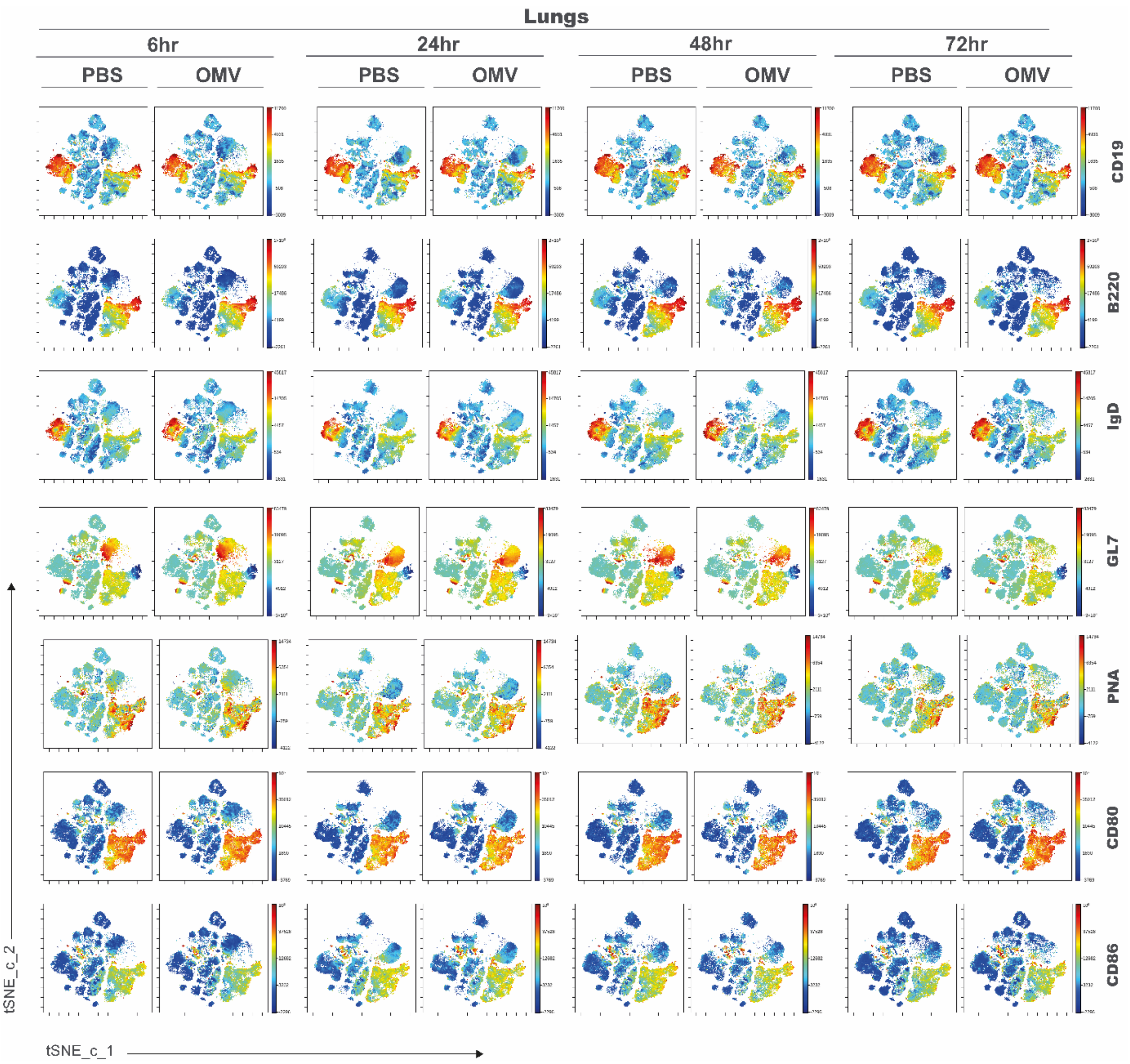

Supplementary figure 4 (continued): t-SNE plots showing marker expression patterns in lungs. t-SNE plots depict the expression profiles of key markers in lungs following OMV vaccination. Plots are concatenated from individual samples (n=5) for each time point and condition. Each marker is displayed with its own independent color scale to indicate relative expression intensity.

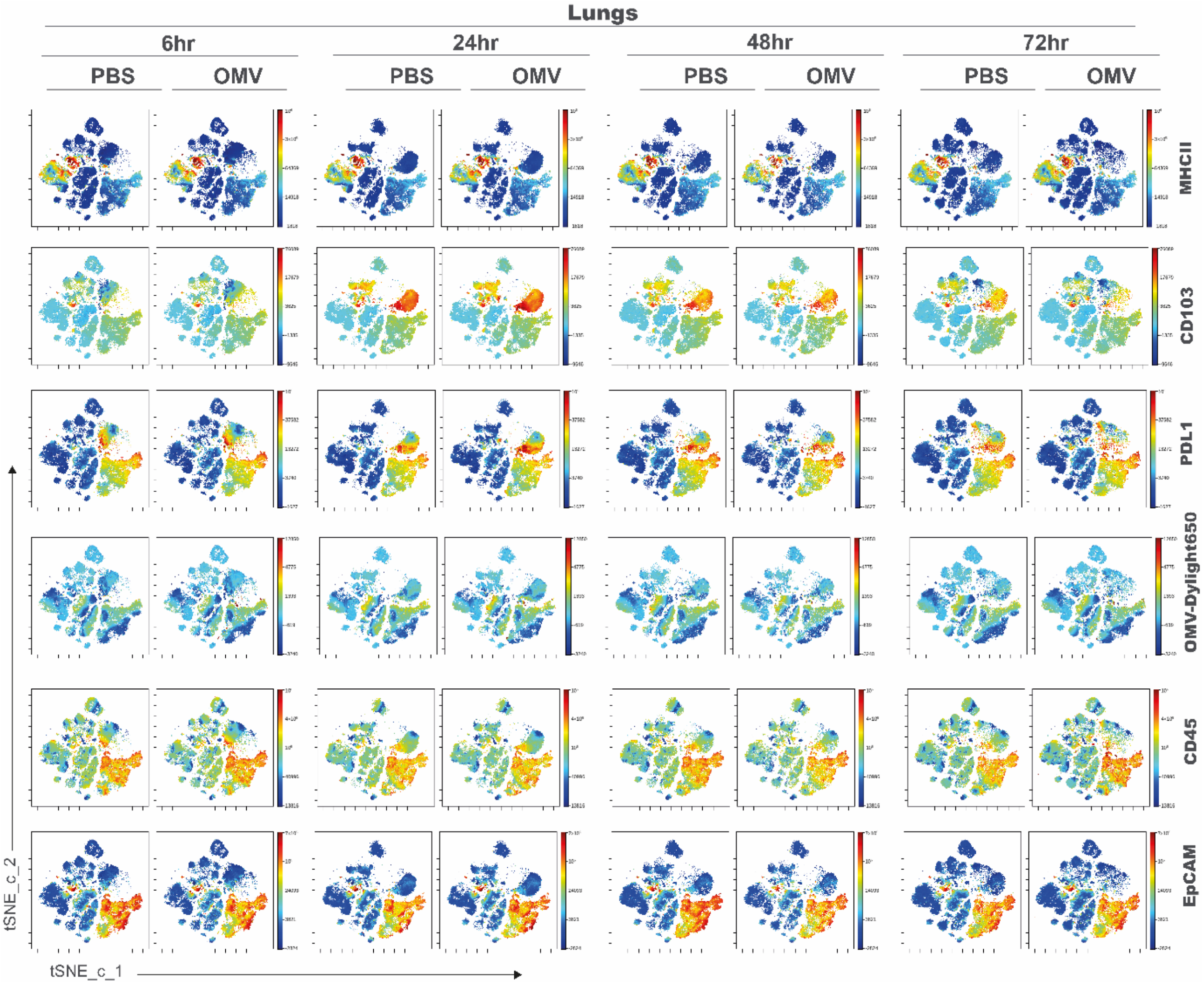

Supplementary figure 4 (continued): t-SNE plots showing marker expression patterns in lungs. t-SNE plots depict the expression profiles of key markers in lungs following OMV vaccination. Plots are concatenated from individual samples (n=5) for each time point and condition. Each marker is displayed with its own independent color scale to indicate relative expression intensity.

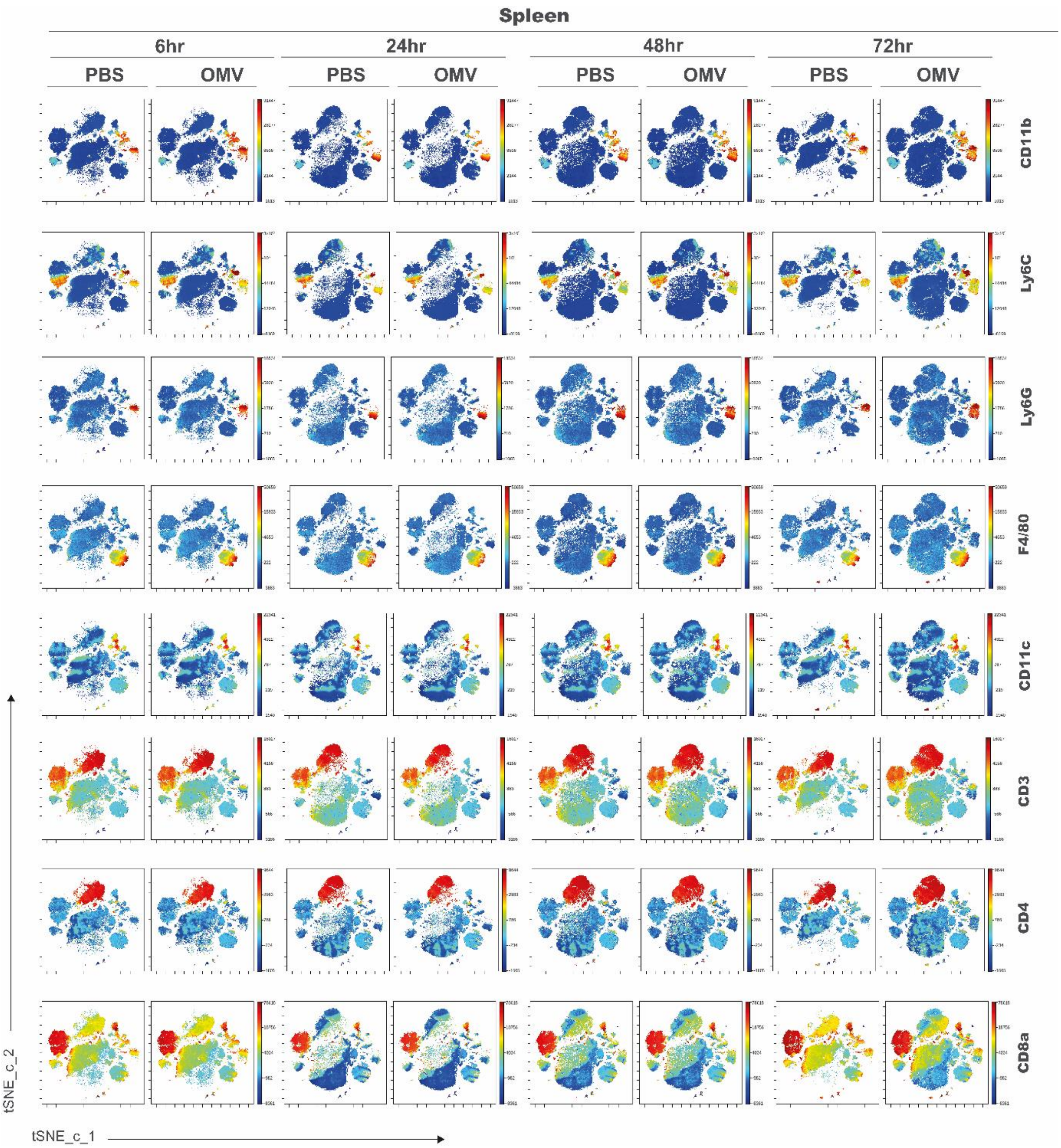

Supplementary figure 5: t-SNE plots showing marker expression patterns in spleen. t-SNE plots depict the expression profiles of key markers in spleen following OMV vaccination. Plots are concatenated from individual samples (n=5) for each time point and condition. Each marker is displayed with its own independent color scale to indicate relative expression intensity.

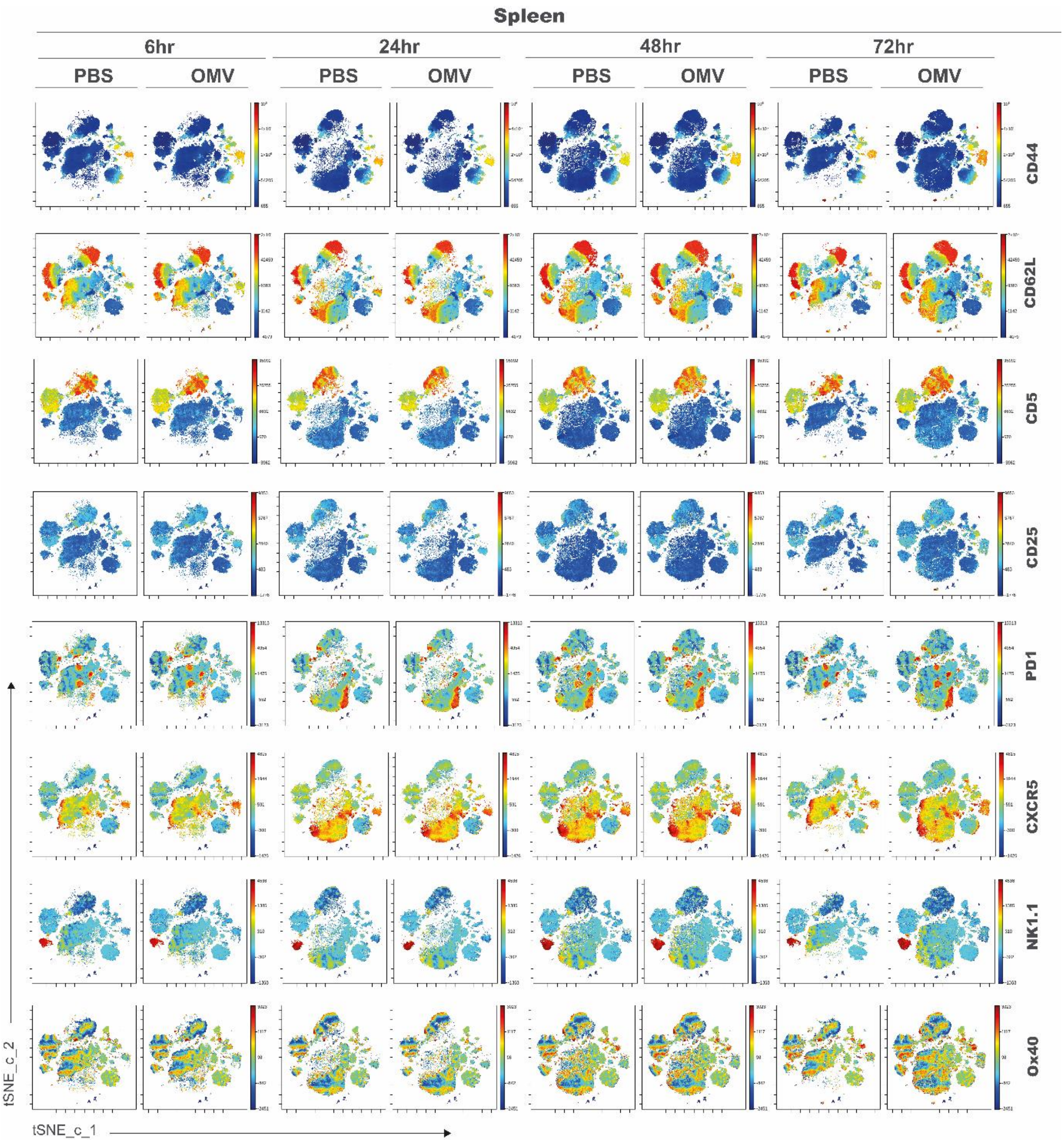

Supplementary figure 5(continued): t-SNE plots showing marker expression patterns in spleen. t-SNE plots depict the expression profiles of key markers in spleen following OMV vaccination. Plots are concatenated from individual samples (n=5) for each time point and condition. Each marker is displayed with its own independent color scale to indicate relative expression intensity.

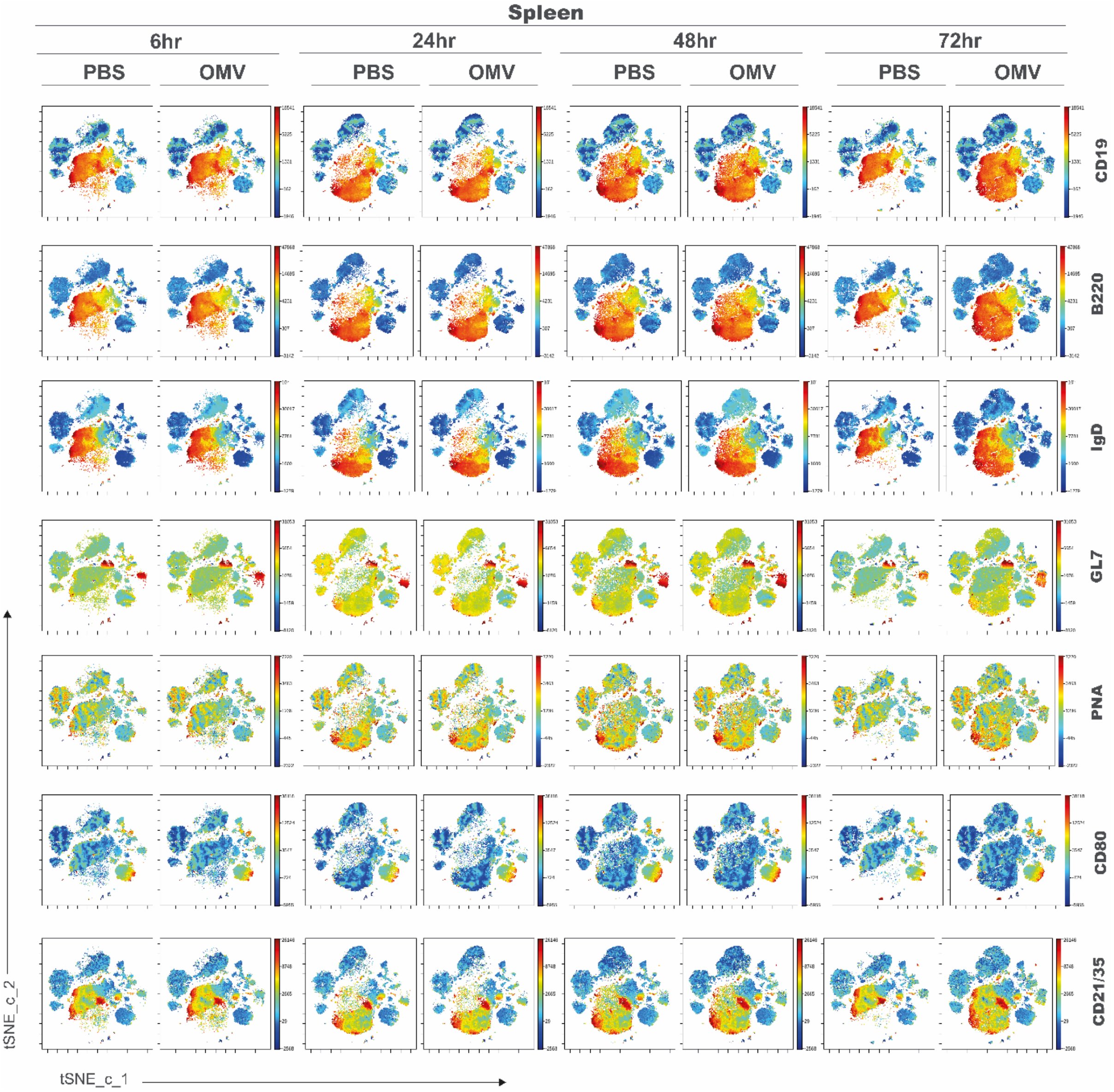

Supplementary figure 5 (continued): t-SNE plots showing marker expression patterns in spleen. t-SNE plots depict the expression profiles of key markers in spleen following OMV vaccination. Plots are concatenated from individual samples (n=5) for each time point and condition. Each marker is displayed with its own independent color scale to indicate relative expression intensity.

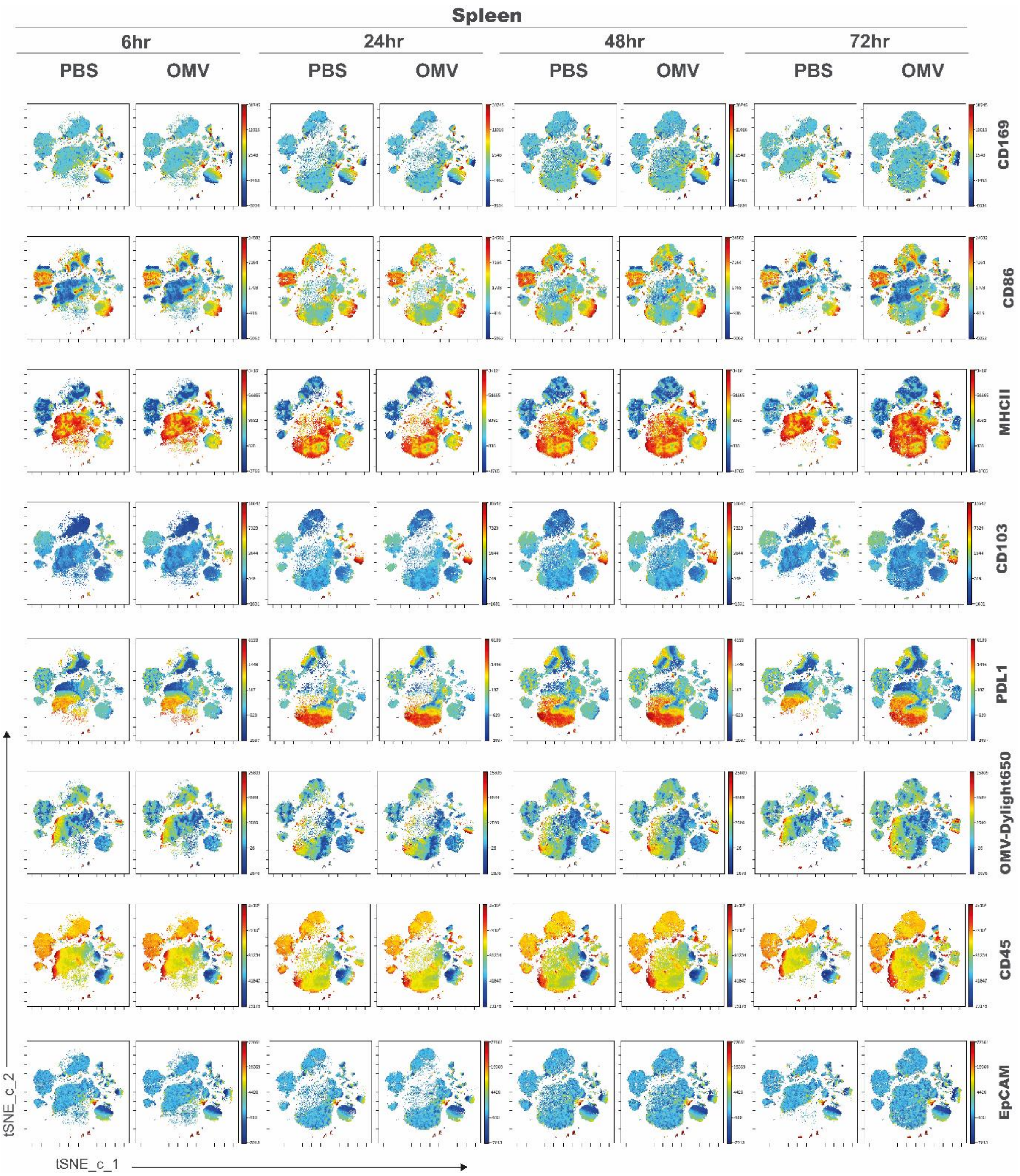

Supplementary figure 5 (continued): t-SNE plots showing marker expression patterns in spleen. t-SNE plots depict the expression profiles of key markers in spleen following OMV vaccination. Plots are concatenated from individual samples (n=5) for each time point and condition. Each marker is displayed with its own independent color scale to indicate relative expression intensity.

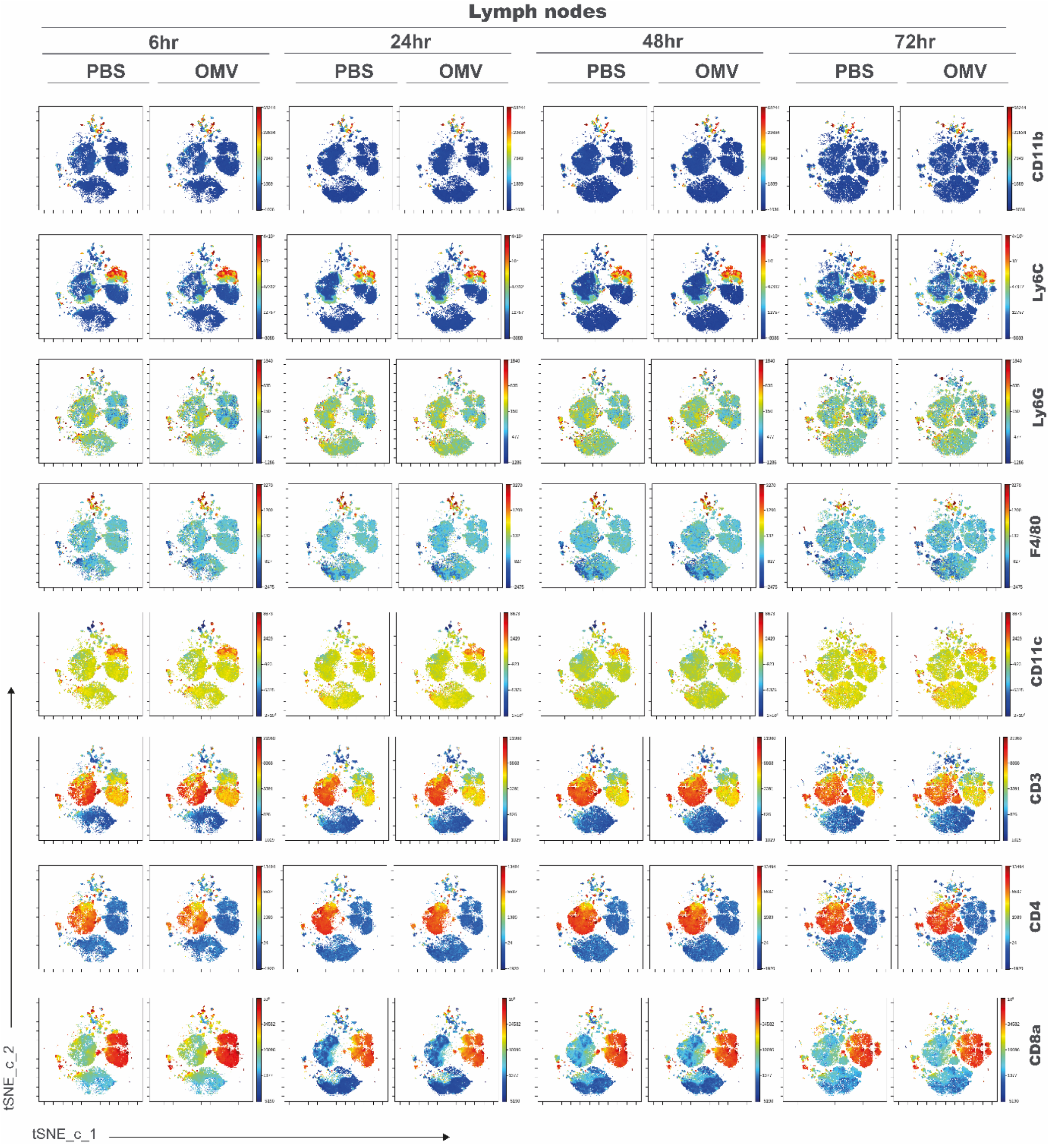

Supplementary figure 6: t-SNE plots showing marker expression patterns in lymph nodes. t-SNE plots depict the expression profiles of key markers in lymph nodes following OMV vaccination. Plots are concatenated from individual samples (n=5) for each time point and condition. Each marker is displayed with its own independent color scale to indicate relative expression intensity.

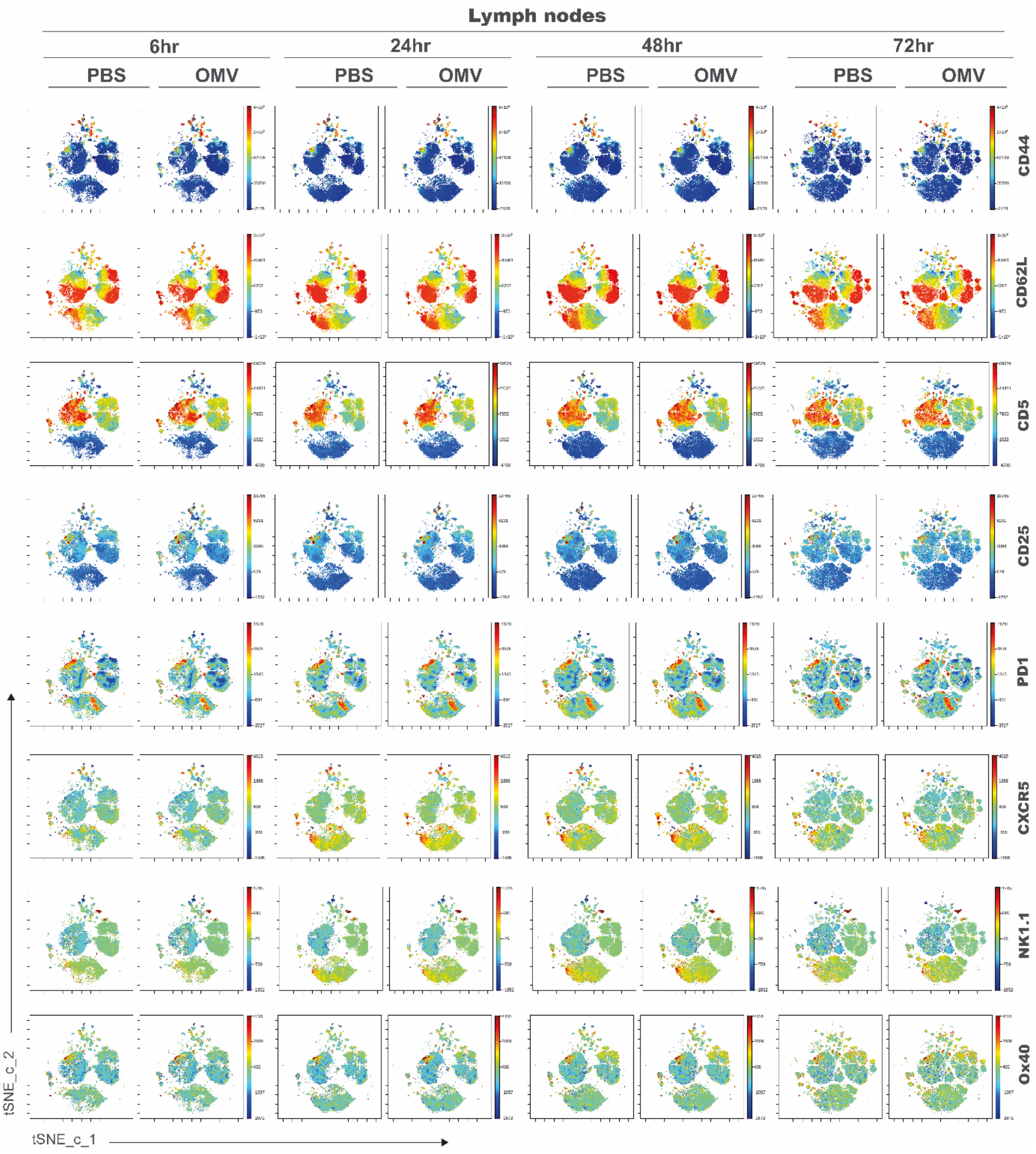

Supplementary figure 6 (Continued): t-SNE plots showing marker expression patterns in lymph nodes. t-SNE plots depict the expression profiles of key markers in lymph nodes following OMV vaccination. Plots are concatenated from individual samples (n=5) for each time point and condition. Each marker is displayed with its own independent color scale to indicate relative expression intensity.

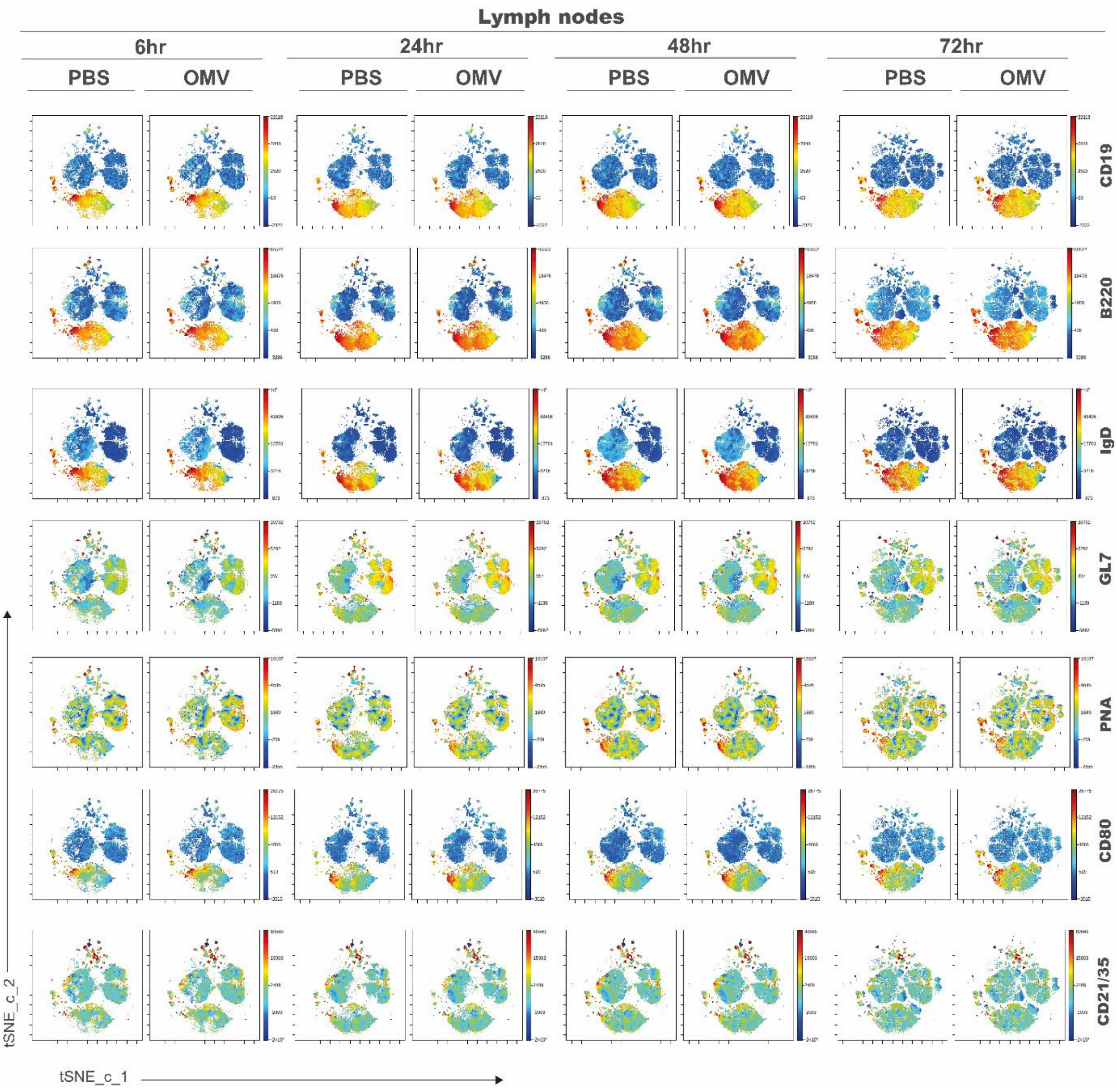

Supplementary figure 6 (Continued): t-SNE plots showing marker expression patterns in lymph nodes. t-SNE plots depict the expression profiles of key markers in lymph nodes following OMV vaccination. Plots are concatenated from individual samples (n=5) for each time point and condition. Each marker is displayed with its own independent color scale to indicate relative expression intensity.

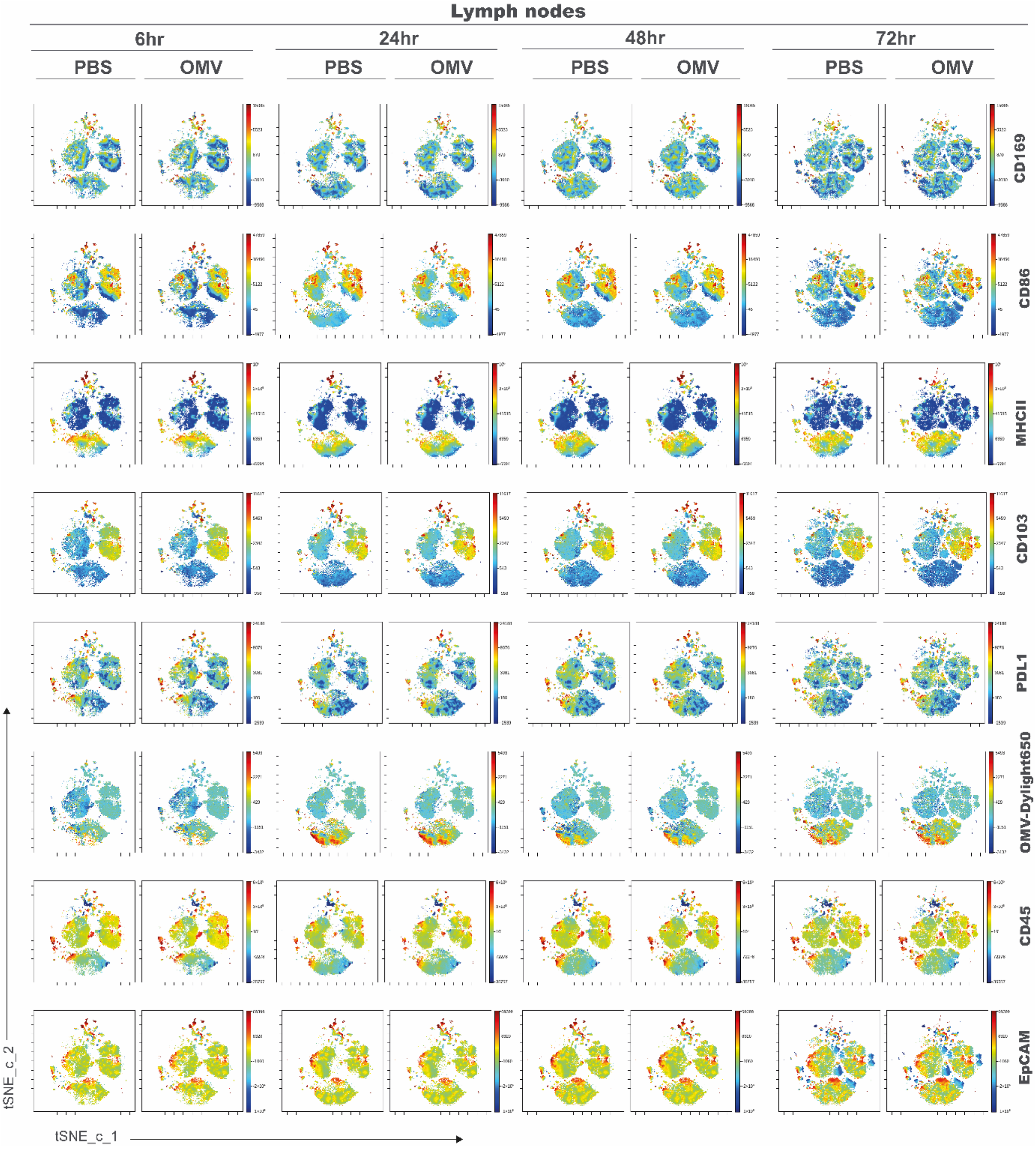

Supplementary figure 6 (Continued): t-SNE plots showing marker expression patterns in lymph nodes. t-SNE plots depict the expression profiles of key markers in lymph nodes following OMV vaccination. Plots are concatenated from individual samples (n=5) for each time point and condition. Each marker is displayed with its own independent color scale to indicate relative expression intensity.

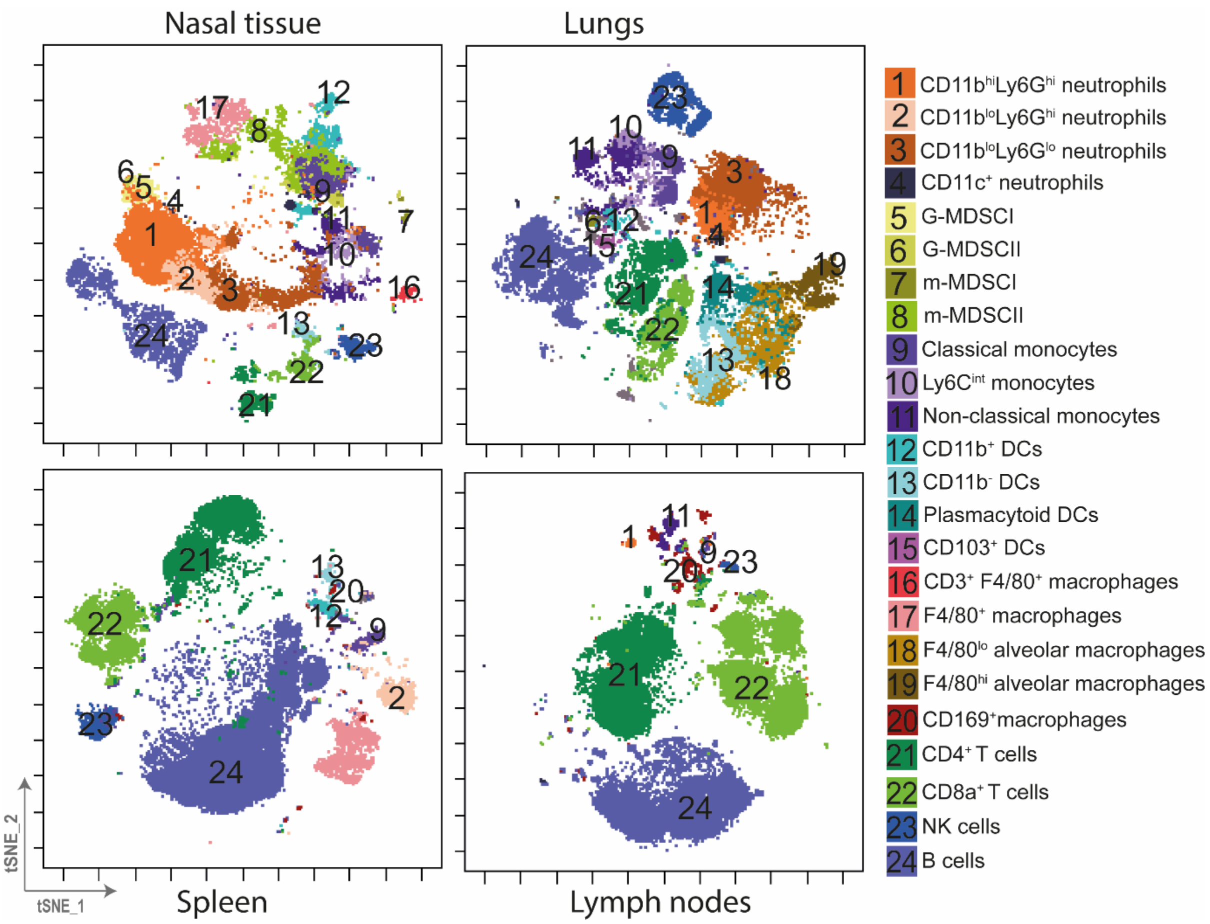

Supplementary figure 7: Representative t-SNE plots showing key immune phenotypes across murine tissues. t-SNE plots depict the major phenotypes identified in nasal tissue, lungs, spleen, and draining lymph nodes following OMV vaccination. Phenotypes were determined by applying FlowSOM clustering analysis to live CD45^{+} immune cells within each tissue.

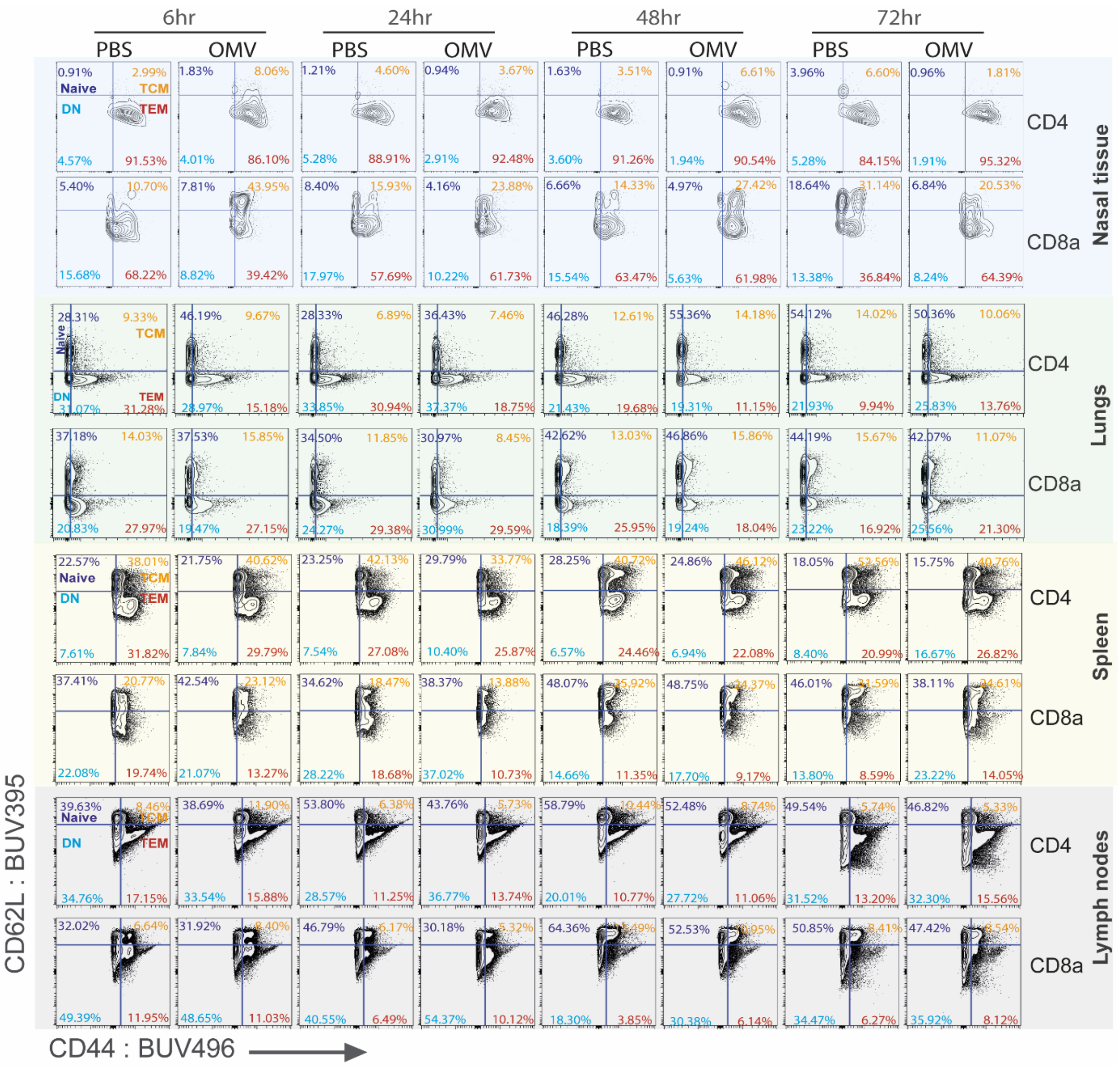

Supplementary figure 8. Contour plots illustrating the spatio-temporal distribution and kinetics of major T cell subtypes post vaccination; contour plots are concatenates of individual samples and percentages represent mean values from individual samples (n = 5).

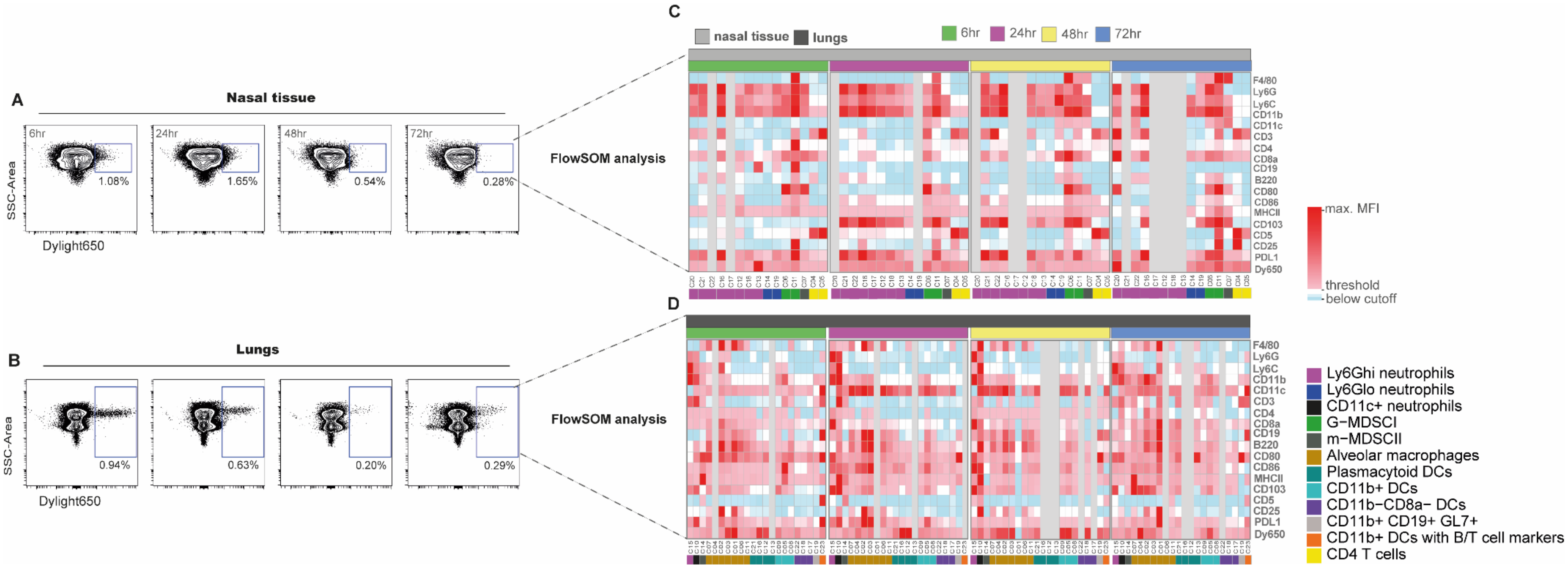

Supplementary figure 9: Characterization of OMV⁺ immune cell populations in nasal tissue and lungs. (A, B) Contour plots showing OMV-positive populations in nasal tissue and lungs, with corresponding mean percentages from concatenated individual samples (n=4–5 per group). **(C, D)** Heatmaps presenting a detailed breakdown of OMV⁺ clusters identified by FlowSOM analysis within the OMV-positive gates from panels A and B. Median fluorescence intensities of respective markers are represented using the indicated color scale. The color panel on the right depicts the phenotype color coding for clusters identified.

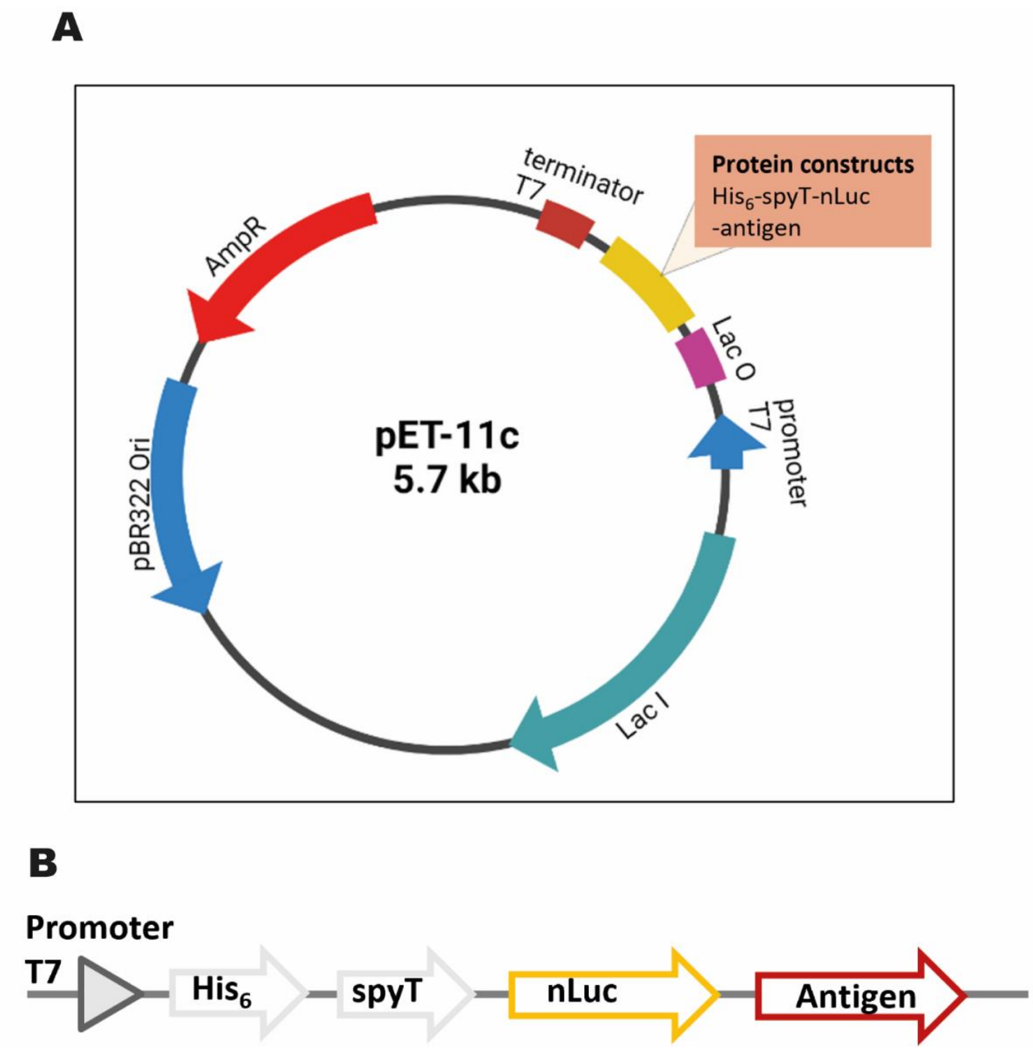

Supplementary figure 10. Design and graphical representation of the nLuc-fusion construct. (a) Representative vector map illustrating the design of the nanoluciferase (nLuc)-fusion construct used for antigen display on OMVs. (b) Graphical schematic showing the structure and components of the nLuc-fusion construct.

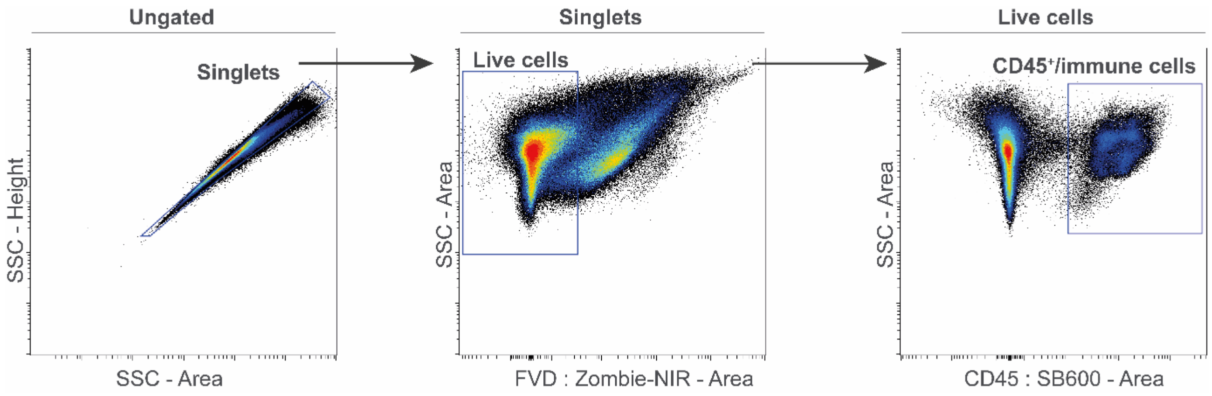

Supplementary figure 11: Gating strategy for spectral flow cytometry analysis. Doublets were excluded using side scatter height (SSC-H) versus side scatter area (SSC-A) gating. Dead cells were removed by negative selection using fixable viability dye (Zombie NIR). Live single cells were then gated for CD45 expression to identify total immune cells. The resulting $CD45^{+}$ cell population was used for unsupervised analysis, including clustering via FlowSOM and dimensionality reduction using tSNE-CUDA.

Supplementary video 1: Sequential 2D slices from a focused ion beam-scanning electron microscopy (FIB-SEM) volume acquisition, visualized as a time-lapse animation to illustrate ultrastructural features within the imaged cell.

Supplementary Table 1: Details of markers and corresponding fluorochromes used in the spectral flow cytometry panel. The table lists all markers included in the flow cytometry panel, specifying target cell populations or purposes, antibody clones, conjugated fluorochromes, and manufacturers.

**Marker**	**Target cells/purpose**	**Clone**	**Fluorochrome**	**Manufacturer**
CD45	Immune cells	30-F11	SB600	Invitrogen (TF)
CD19	B-cells	6D5	BUV750	Biolegend
CD45R/B220	B-1 cells	RA1-6B2	Spark violet 538	Biolegend
IgD	Immature B cells	11-26c.2a	APC	Biolegend
GL7	GC B cells	GL7	PE	Biolegend
biotin-PNA	GC B cells	x	x	Vectorlabs
CD21/35	Follicular dendritic cells, B cells	7G6 (RUO)	BUV805	BD Optibuild
CD3	T cells	145-2C11	BV785	BioLegend
CD4	CD4+ T cells	GK1.5 (RUO)	APC-Cy7	BD Pharmingen
CD8a	CD8+ T cells	QA17A07	Spark YG 593	Biolegend
CD62L	Naive and effector memory T cells	MEL-14	BUV395	BD Horizon
CD44	Naïve and effector memory T cells	IM7 (RUO)	BUV496	BD Optibuild
CD5	T cell activation marker	53-7.3	BUV661	BD Optibuild

CD25	Treg/T cell activation marker	PC61 (RUO)	BUV737	BD Horizon
CXCR5	Follicular helper T cells	L138D7	PerCP5.5	Biolegend
PD1	T Follicular helper T cells	29F.1A12	PE-Fire640	Biolegend
Ox40	Follicular helper T cells	OX-86	BV711	Biolegend
CD11b	Myeloid cells	M1/70 (RUO)	PE-Cy7	BD Pharm.
Ly6C	Neutrophils, Monocytes	HK1.4	BUV615	BD Optibuild
Ly6G	Neutrophils	1A8	PE-Fire810	BioLegend
F4/80	Macrophages	BM8	BUV563	Thermoscientific
CD11c	Dendritic cells	N418	Spart Red 718	Biolegend
CD169	Macrophages (lymphoid tissues)	3D6.112	FITC	Biolegend
CD80	Activation marker	16-10A1	PE/Cy5	Biolegend
CD86	Activation marker	GL-1	PE/Dazzle 594	Biolegend
MHCII	Activation marker	AF6-120.1 (RUO)	BV421	BD Horizon
CD103	Homing marker, tissue residence	QA17A24	PerCP/Fire™ 780	Biolegend
PDL1	Exhaustion marker for myeloid cells	10F.9G2	BV650	Biolegend

Streptavidin	for PNA (biotin)	x	PerCP	Biolegend
OMVs	Vaccine particles	x	AF647	Aberabiosciences
Fixable viability dye (FVD)	dead cells	x	Zombie NIR	Biolegend

TableS2: Amino acid sequences of His_6-tagged nanoluciferase-fused proteins. The table lists the full amino acid sequences of His_6-tagged nanoluciferase (nLuc) fusion proteins used for OMV surface display, including constructs for SpyTag-nLuc, SpyTag-nLuc-PnrA, and SpyTag-nLuc-AliA, with respective sequence lengths, molecular weights, and isoelectric points.

Histidine tag<br>SpyTag<br>nLuc	>SpT-nLuc-protein (218 aa) 23.79kDa<br>MGSSHHHHHHSSGLVPRGSHMGVPTIVMVDAYKRYKGSGGSGVFTLEDFVGDWRQTAGYNLDQVLEQGGVSSLFQ<br>NLGVSVTPIQRIVLSGENGLKIDIHVIIPYEGLSGDQMGQIEKIFKVVYPVDDHHFKVILHYGTLVIDGVTPNMIDYFGRPYE<br>GIAVFDGKKITVTGTLWNGNKIIDERLINPDGSLLFRVTINGVTGWRLCERILAGSGGTG
Histidine tag<br>SpyTag<br>nLuc<br>PnrA	>SpT-nLuc-PnrA protein (546 aa) 58.33 kDa pI 5.77<br>MGSSHHHHHHSSGLVPRGSHMGVPTIVMVDAYKRYKGSGGSGVFTLEDFVGDWRQTAGYNLDQVLEQGGVSSLFQ<br>NLGVSVTPIQRIVLSGENGLKIDIHVIIPYEGLSGDQMGQIEKIFKVVYPVDDHHFKVILHYGTLVIDGVTPNMIDYFGRPYE<br>GIAVFDGKKITVTGTLWNGNKIIDERLINPDGSLLFRVTINGVTGWRLCERILAGSGGTGGNRSSRNAASSSDVKTKAAIV<br>TDTGGVDDKSFNQSAWEGLQAWGKEHNLSKDNGFTYFQSTSEADYANNLQQAAGSYNLIFGVGFALNNAVKDAAKE<br>HTDLNYVLIDDVIKDQKNVASVTFADNESGYLAGVAAAKTTKTKQVGFVGGIESEVISRFEAGFKAGVASVDPSIKVQVD<br>YAGSFGDAAKGKTIAAAQYAAGADIVYQVAGGTGAGVFAEAKSLNESRPENEKVWVIGVDRDQEAEGKYTSKDGKESN<br>FVLVSTLKQVGTTVKDISNKAERGEFPGGQVIVYSLKDKGVDLAVTNLSEEGKKAVEDAKAKILDGSVKVPEK
Histidine tag<br>SpyTag<br>nLuc<br>AliA	>SpT-nLuc-AliA protein (855 aa) 64.59kDa pI 5.27<br>MGSSHHHHHHSSGLVPRGSHMGVPTIVMVDAYKRYKGSGGSGVFTLEDFVGDWRQTAGYNLDQVLEQGGVSSLFQ<br>NLGVSVTPIQRIVLSGENGLKIDIHVIIPYEGLSGDQMGQIEKIFKVVYPVDDHHFKVILHYGTLVIDGVTPNMIDYFGRPYE<br>GIAVFDGKKITVTGTLWNGNKIIDERLINPDGSLLFRVTINGVTGWRLCERILAGSGGTGSGSGSSTKGEKTFSYIYETDPD<br>NLNYLTTAKAATANITSNVVDGLLENDRYGNFVPSMAEDWSVSKDGLTYTYTIRKDAKWYTSEGEEYAAVKAQDFVTGL<br>KYAADKKSDALYLVQESIKGLDAYVKGEIKDFSQVGIKALDEQTVQYTLNKPESFWNSKTTMGVLAPVNEEFLNSKGDDF<br>AKATDPSSLLYNGPYLLKSIVTKSSVEFAKNPNYWDKDNVHVDKVKLSFWDGQDTSKPAENFKDGSLTAARLYPTSASFA<br>ELEKSMKDNIVYTQQDSITYLVGTNIDRQSYKYTSKTSDEQKASTKKALLNKDFRQAIAFGFDRTAYASQLNGQTGASKIL<br>RNLFVPPTFVQADGKNFGDMVKEKLVTYGDEWKDVNLADSQDGLYNPEKAKAEFAKAKSALQAEGVQFPIHLDMPVD<br>QTATTKVQRVQSMKQSLEATLGADNVIIDIQQLQKDEVNNITYFAENAAGEDWDLSDNVGWGPDFADPSTYLDIIKPS<br>VGESTKTYLGFDSGEDNVAAKKVGLYDYEKLVTEAGDETTDVAKRYDKYAAAQAWLTDSALIIPTTSRTGRPILSKMVPFT<br>IPFALSGNKGTSEPVLYKYLELQDKAVTVDEYQKAQEKWMKEKEESNKKAQEDLAKHVK
